# Fast Pyrolysis Behavior of Banagrass as a Function of Temperature and Volatiles Residence Time in a Fluidized Bed Reactor

**DOI:** 10.1371/journal.pone.0136511

**Published:** 2015-08-26

**Authors:** Trevor James Morgan, Scott Q. Turn, Anthe George

**Affiliations:** 1 Hawaii Natural Energy Institute, University of Hawaii at Manoa, Honolulu, HI, 96822, United States of America; 2 Combustion Research Facility, Sandia National Laboratories, Livermore, California, 94550, United States of America; INSTITUTO MEXICANO DEL PETRÓLEO, MEXICO

## Abstract

A reactor was designed and commissioned to study the fast pyrolysis behavior of banagrass as a function of temperature and volatiles residence time. Four temperatures between 400 and 600°C were examined as well as four residence times between ~1.0 and 10 seconds. Pyrolysis product distributions of bio-oil, char and permanent gases were determined at each reaction condition. The elemental composition of the bio-oils and chars was also assessed. The greatest bio-oil yield was recorded when working at 450°C with a volatiles residence time of 1.4 s, ~37 wt% relative to the dry ash free feedstock (excluding pyrolysis water). The amounts of char (organic fraction) and permanent gases under these conditions are ~4 wt% and 8 wt% respectively. The bio-oil yield stated above is for 'dry' bio-oil after rotary evaporation to remove solvent, which results in volatiles and pyrolysis water being removed from the bio-oil. The material removed during drying accounts for the remainder of the pyrolysis products. The 'dry' bio-oil produced under these conditions contains ~56 wt% carbon which is ~40 wt% of the carbon present in the feedstock. The oxygen content of the 450°C, 1.4 s 'dry' bio-oil is ~38 wt%, which accounts for ~33 wt% of the oxygen in the feedstock. At higher temperature or longer residence time less bio-oil and char is recovered and more gas and light volatiles are produced. Increasing the temperature has a more significant effect on product yields and composition than increasing the volatiles residence time. At 600°C and a volatiles residence time of 1.2 seconds the bio-oil yield is ~21 wt% of the daf feedstock, with a carbon content of 64 wt% of the bio-oil. The bio-oil yield from banagrass is significantly lower than from woody biomass or grasses such as switchgrass or miscanthus, but is similar to barley straw. The reason for the low bio-oil yield from banagrass is thought to be related to its high ash content (8.5 wt% dry basis) and high concentration of alkali and alkali earth metals (totaling ~2.8 wt% relative to the dry feedstock) which are catalytic and increase cracking reactions during pyrolysis.

## Introduction

The Hawaii Clean Energy Initiative [1] includes ongoing assessment of renewable energy opportunities in the State, including thermochemical and biological conversion of biomass, wind, ocean, geothermal, and solar. This manuscript present results from the fast pyrolysis of a tropical biomass species, banagrass (*Pennisetum purpureum x Pennisetum glaucum*), which is of interest as a potential energy crop in Hawaii and the tropics. Banagrass is a fast growing species that can be grown in quantities of 40 to 65 Mg fiber ha^-1^ yr^-1^ with nonirrigated and irrigated productivities representing the lower and higher end of this range, respectively [2–4]. The higher heating value (HHV) of banagrass is ~17 MJ/kg which makes it an interesting prospect when searching for nonfood crops suitable for energy applications. For context, switchgrass can typically be grown in quantities of 5–15 Mg fiber ha^-1^ yr^-1^ and has a HHV of ~18.5 MJ/kg [5].

Fast pyrolysis is a potential pathway for producing significant quantities of renewable liquid fuels, often referred to as bio-liquids or bio-oils, from lignocellulosic biomass materials [6, 7]. Much of the ground work on biomass pyrolysis dates back to studies by Shafizadeh [8, 9] and Scott [10, 11] in the 1970's. With significant contributions from Elliot [12], Kandiyoti [6], Antal [13, 14], Diebold [15, 16], Oasmaa [17, 18] and Czernik [15, 19], throughout the 1980's and 1990's. Recent review articles have detailed the progress up to recent times [6, 7, 20].

Fast pyrolysis is of interest as pyrolysis oils have lower activation energies than the parent solid fuel making combustion easier and more efficient [21]. In addition, bio-oils have greater fuel densities than biomass which makes transportation less costly. Raw bio-oil is reported to be a suitable replacement for fuel oils used in boiler and furnace applications [22]. This is of particular interest in Hawaii where low sulfur fuel oil is the main source of electricity production. Raw bio-oil, however, has high acidity which makes storage and transportation an issue. It also has high oxygen content which lowers its heating value compared to traditional oils and it is also reactive, i.e. aging is a problem. Hence there is great interest in upgrading raw bio-oils to create a more stable product and to produce transportation fuels (diesel, gasoline and jet-fuel). At present upgrading is not cost effective due to catalyst deactivation problems and excessive coke and CO_2_ production [6, 7, 20].

The fast pyrolysis of woody biomass, grasses and agricultural residues have been widely studied [22–26]. Typically woody biomass (e.g. eucalyptus, pine, beech) gives the greatest bio-oil yield, up to ~65–75 wt% of the dry ash free (daf) feedstock [22–25]. Grasses (switchgrass, miscanthus) tend to produce slightly less bio-oil than woods, typical bio-oil yields from grasses are ~50–60 wt% (daf feedstock) [23, 24, 26]. Agricultural residues (wheat straw, barley straw) tend to produce the least bio-oil ~30–50 wt.% (daf feedstock) [22–24]. There can be, however, significant variations in product distributions for the same species depending on growing conditions. The main causes for differences in product distributions and product quality is related to the chemical composition of the biomass, i.e. cellulose, hemicellulose and lignin contents (and type of lignin). More important, however, is the ash composition and quantity [22–24, 27]. The significant role of inorganics (ash) on biomass pyrolysis has been reported upon since the 1980's [9, 11]. High concentrations of alkali and alkali earth metals (AAEM) increase/catalyze cracking reactions and reduce bio-oil and possibly char yields, and affect oil quality.

Despite their tendency to produce less bio-oil then woody species, grasses are of interest as pyrolysis feedstocks because they are fast growing and can be grown on lands unsuitable for food crops [22, 23]. The reason for the lower bio-oil yields from grasses than woody biomasses is mostly related to their high ash concentrations, in particular due to AAEM. A review of the composition of ~80 biomass species has recently been reported [28, 29].

Banagrass is a species of elephant grass (*Pennisetum purpureum Schum*) which is similar to napier grass (*Pennisetum purpureum*). While there are a great number of publications examining the yields and conditions under which these grasses can be grown, there are few reports on their pyrolysis behavior. Banagrass has been examined in air blown gasification studies [30] and for metallurgical biocarbon production [4]. However, there are no reports (to our knowledge) on the fast pyrolysis of *Pennisetum purpureum* species for bio-oil production. Mesa-Perez et al. [31] examined the fast pyrolysis of elephant grass in a 200 kg/h pilot plant for charcoal production but bio-oil and gas yields were not given. Slow pyrolysis studies of elephant grass have been reported using fixed-bed reactor configurations [32, 33]. Braga et al. [34] reported on the slow pyrolysis of elephant grass (*Pennisetum purpureum Schum*) after pretreatment via hot water or acid washing. Washing reduced the ash content from ~7.0 wt% wet basis to ~2.5 wt% and improved the volatiles yield and apparently reduced activation energy. However, the study was carried out using a thermogravimetric analyzer and the ash composition was not reported making their findings difficult to interpret.

Two slow pyrolysis studies of napier grass have been reported. Mythili et al. [35] obtained ~26 wt% bio-oil and ~28 wt% char (basis not given) using a fixed bed reactor with a maximum heating rate of 15°C/min to 450°C. Lee et al.[36] reported a maximum bio-oil yield of ~35 wt% with ~30 wt% char (basis not given) using a heating rate of 150°C/min to 500°C. In both studies the residence times of volatile products was not reported and it is not clear whether the bio-oil yields include pyrolysis water and / or moisture.

Achieving high biomass carbon conversion and high bio-oil yields while maintaining bio-oil quality requires a balance between heating rate, maximum temperature, and vapor residence time. Typical conditions for biomass fast pyrolysis are characterized by heating rates in excess of ~300°C/s, vapor residence time of ~1 second and a pyrolysis temperature in the range of 400 to 500°C. For any particular feedstock, the reactor configuration chosen to perform fast pyrolysis has a significant influence on product yields, distribution, and chemical composition of the char, oil and permanent gases. For example, fixed bed reactors produce more char and less oil than fluidized bed designs and there can be significant variations to product yield given different fluidized bed designs and due to reactor scale [6, 37]. The various approaches that have been developed for the pyrolysis of biomass and coal have been detailed in review articles [6, 7, 20]. Differences in products yields and composition mentioned above are related to secondary reactions of the primary species that occur in the bed and freeboard during pyrolysis. If secondary reactions in the pyrolysis process are not controlled through reactor design and initial fuel particle size selection, the results can be inconsistent and misleading, as described elsewhere [6, 14].

Therefore to assess the fast pyrolysis behavior of a solid fuel or when generating comparative data sets from a number of different fuels it is important to reduce the influence of uncontrolled secondary reactions on the results as much as is practically possible. This can be achieved by rapidly cooling and condensing the pyrolysis vapors, and by grinding the feedstock as finely as practically possible (i.e. to between 100 and 200 μm [6, 21]) to minimize the contact between escaping vapors and the solid particle mass. When the aim of pyrolysis is to maximize the bio-oil yield it is also beneficial to reduce particle—particle interactions through the use of a fluidized bed or entrained flow type of reactor design. In this way reliable trends can be obtained for the pyrolysis behavior of the feedstock. In addition, the data can be used to eliminate feedstocks, processes, or process conditions that are unlikely to prove feasible for large scale, economically viable applications. These issues have been described in detail elsewhere [6, 7].

Based on designs reported in the literature [21, 38], a fluidized bed reactor was constructed that included the ability to alter the position of the bed support plate so that volatiles’ residence times could be varied without changing the fluidizing conditions. Results are presented, below, for the fast pyrolysis behavior of banagrass over four residence times (between ~1.0 and 10.0 seconds) and at four temperatures (between 400 and 600°C). In addition, results from commissioning tests with cellulose are reported to assess the performance of the newly constructed reactor system. Product distribution (amounts of char, bio-oil and gas) results are presented for each reaction condition. The carbon, hydrogen, oxygen, and nitrogen contents of bio-oil, as determined by combustion analysis, were used as indicators of bio-oil quality. The bio-oil samples were examined by GC-MS.

## Experimental

### Sample preparation

Cellulose powder (MP biomedicas, LCC, USA. Part number: 191499, CAS: 9004-34-6) with an mean particle size of ~100 μm, a moisture content of 2.0 wt% and ash content of 0% was used in the condition it was received.

Approximately 100 kg of ten month old banagrass was harvested from the Waimanalo Experiment Station of the University of Hawaii. The above ground material was processed into nominal 50 mm pieces (stalk and leaves) using a shredder (Vincent Corp., Tampa, FL) and dried to equilibrium moisture content (~10% moisture) using an ambient air drying bed. A representative sub sample of ~200 g was ground to particle sizes that passed through a screen with a 200 μm opening. Examination of the ground sample under a microscope revealed that approximately 60–70% of the sample was a powder with a particle size in the range of 100 to 200 μm and ~30–40% was rod-like with a diameter <200 μm and a length of ~400 to 600 μm. The proximate analysis (ASTM D 3172), ultimate analysis (ASTM D 3176) and ash composition (ASTM D3682, 600°C) of banagrass were determined at an accredited laboratory (Hazen Research Inc., Golden, CO).

Total carbohydrate and lignin content (compositional analysis) of banagrass was measured in triplicate using the National Renewable Energy Laboratory procedure TP-510-42618 and instrumentation described elsewhere [39, 40]. The relative standard deviation of the results from compositional analysis are <2.0%.

### Fluidized bed reactor

**Fig 1:**
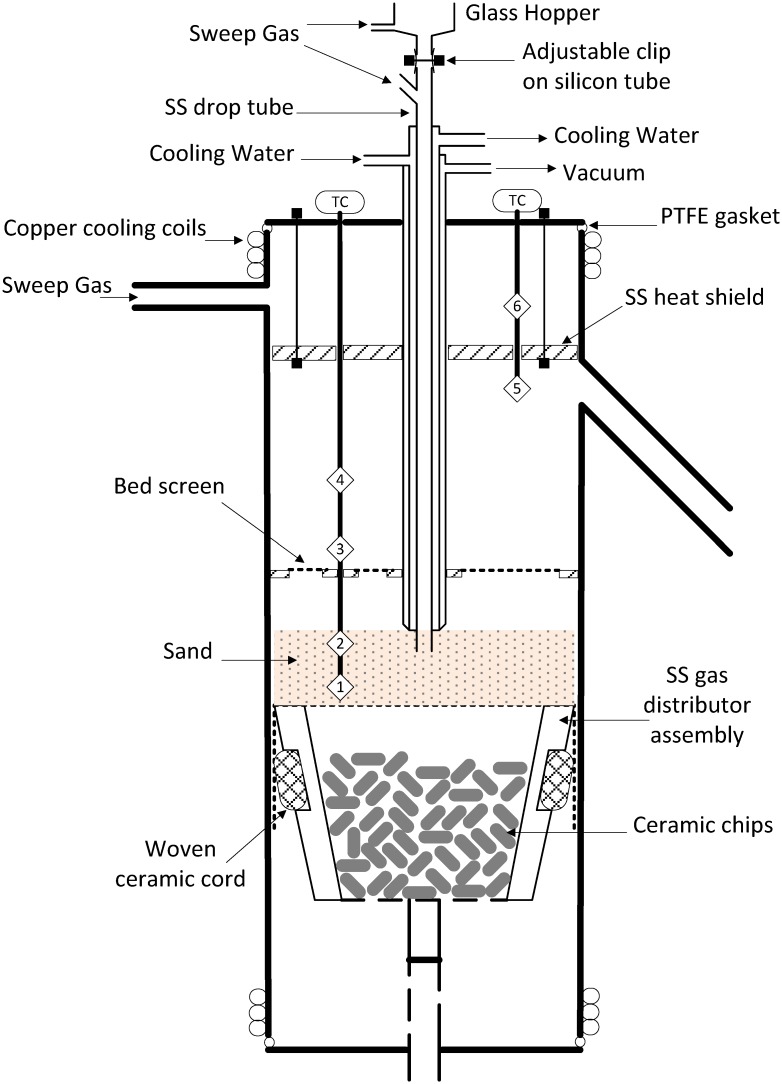
Schematic diagram of the variable-freeboard pyrolysis reactor. Numbers 1 through 6 show the locations of the thermocouples in the multi-point temperature probes (SS—stainless steel).

A fluidized bed reactor was constructed and used for the first time in the present study. Many aspects of the design were adapted from Stiles [21, 38] including an inert sand fluidized bed, a moveable bed support plate, a mesh screen to retain char and fines in the bed, and a side-arm for volatiles removal from the free-board (Fig 1). Approximately 500 g of fresh sand was used as bed material for each experiment (Acros Organics, USA, acid washed sand, 40–100 mesh, mean particle size 205 μm, code number: 37094–0000, CAS: 14808-60-7). The elemental composition of the sand is SiO_2_: 99.8%, Fe_2_O_3_: 0.009%, Al_2_O_3_: 0.040%, TiO_2_: 0.016%, K_2_O: 0.006%, CaO: 0.005%, Cr_2_O_3_: 0.00005% (analysis provided by the supplier).

Three mass flow controllers are used to meter nitrogen to 1) fluidize the bed, 2) entrain feedstock from the fuel hopper into the bed and 3) sweep hot volatiles out of the top of the reactor. The latter helps protect the PTFE gaskets, used to seal the top of the reactor, from exceeding material temperature limits. Fluidization velocities of 2.75 to 3.75 times the minimum fluidization velocity were used. The nitrogen flow used to fluidize the bed is 7.5 LPM (STP) in all cases with an additional 1.5 LPM (STP) entering the top of the bed from the feeder drop-tube. Four bed positions were used to produce different volatiles residence times without altering the fluidizing conditions; corresponding residence times (RT) at each bed position (BP) and temperature are given in Table 1 along with the flow rates required to achieve the minimum fluidization velocity.

**Table 1:** Volatiles residence times (seconds) at the working velocity and flow rates (LPM, STP) to achieve minimum fluidization velocity, for the four different bed positions and four temperatures used in this study. The times are derived from the volume of the freeboard alone, excluding the side-arm where the volatiles pass to the bio-oil traps.

Temperature	Flow rate	Bed Position			
°C	SLPM	BP-1	BP-2	BP-3	BP-4
		Seconds			
400	~2.7	12.2	8.3	4.6	1.5
450	~2.5	11.3	7.7	4.2	1.4
500	~2.3	10.6	7.2	4.0	1.3
600	~2.0	9.4	6.4	3.5	1.2

The reactor body is constructed from 316 stainless steel pipe with inner diameter of 82.8 mm, outer diameter of 88.9 mm, and length of 1.57 m. The central 1.22 m of the reactor body is heated by the furnace and 18.5 cm protrude from either end. A stainless steel water cooled sample drop-tube with an additional, outer, vacuum jacket was used to transfer the feedstock from a glass hopper into the bed. The heating rate of the feedstock as it enters the bed is estimated to be ~400°C/s.

A wire-mesh screen attached to the drop tube, denoted as the 'bed screen,' is located ~5 cm above the stationary bed to prevent losses of char from the bed (see Fig 1). An additional wire mesh screen was placed in the flange gasket that connects the exit side-arm to the first bio-oil trap. The side-arm is heat traced from the exit of the furnace to the flange to maintain a gas temperature of 340–360°C. The flange connecting the side arm and the traps is cooled with dry-ice to quench the exiting vapors and collect them in two, liquid-nitrogen cooled, stainless steel traps in series. A slip-stream of the permanent gases exiting the second bio-oil trap passes through a train of on-line gas analyzers.

Residence times are based on the dimensions of the freeboard and do not include the time taken for the vapors to pass through the side-arm to the bio-oil traps. Including the side-arm volume increases the RT by ~0.5 seconds. A more complete description of the fluidized bed reactor is given in S1 File.

### Experimental procedure

Approximately 7.5 g of feedstock was used for each experiment and took 1.5 to 2.5 minutes to feed into the reactor. Bio-oil was recovered from the traps by washing with a mixture of 80 vol. % acetone and 20 vol. % methanol (HPLC grade, Fisher Chemicals). Samples of the bio-oil solutions (trap-1 and trap-2) were analyzed separately by GCMS. The two bio-oil solutions were stored overnight at -20°C. A rotary evaporator operating at 55°C with a nitrogen purge and a maximum vacuum of ~25" Hg was used to remove the solvent. Three sub-samples from each bio-oil solution were dried and the mean of these determinations is defined here as the 'dry' bio-oil yield. Repeatability of the dry bio-oil yield was assessed by repeating the experiment three times, producing bio-oil solutions from the two oil traps from each experiment, and then sub-sampling and analyzing each oil trap solution three times. In total, three experiments produced a total of eighteen dry bio-oil samples. From this, a standard deviation of ≤2 wt% (absolute) of the daf feedstock was determined. The bias is discussed in the results section.

A sample of the dry bio-oil was dissolved in fresh solvent and analyzed by GCMS. Comparing this dry bio-oil analysis with the analysis of bio-oil solutions before they were dried provides an estimate of the ‘volatile bio-oil’ fraction removed with the solvent during rotary evaporation. The repeatability and bias of the 'volatile bio-oil' yield is discussed in the GCMS experimental section. Rotary evaporation resulted in water being lost from the bio-oil samples, therefore determination of pyrolysis water was not attempted.

The repeatability of the char yield determinations was ~±1.5 wt% (absolute). The bias in the char yield is estimated to be ≤±2.0 wt% (absolute). Char samples were ashed in a muffle furnace at 600°C, accordingly, char yields were corrected to a dry ash free (daf) basis. Char yields are reported for the organic fraction (Char_Org_) excluding ash, i.e. on a daf basis relative to the daf feedstock. Char yields are also reported inclusive of ash (Char_Org+Inorg_) on a dry basis relative to the dry feedstock. A more detailed account of the experimental procedure is provided in S2 File.

### Uncertainty, Repeatability and Bias

The terminology used to discuss uncertainty associated with the pyrolysis experiments are defined as follows. The repeatability (or precision) of the results is assessed in terms of the standard deviation which is derived from repeated measurement of the same sample under the same conditions. The bias refers to an estimate of the accuracy of the results based on an assessment of potential sources of losses throughout the various procedures, as well as referring to systematic uncertainties in the data which are estimated through comparison with literature values, where possible.

### GCMS

A Bruker SCION GCMS with a BPx-1701 column (Restek corp., USA. 60 m x 0.25 mm, x 0.10 μm) was used to analyze the bio-oils. The analysis used an injector temperature of 280°C. The column temperature was held at 40°C for 4 minutes, ramped to 280°C at 3°C/min and then held at 280 for 20 minutes. The conditions are based on those reported by Mohan et al. [41]. A certified standard (Restek corp., USA) was used to produce a calibration curve for 17 individual compounds: cyclohexane (CAS 110-82-7), furfural (CAS 98-01-1), 3-methyl-2-cyclopenten-1-one (CAS 2758-18-1), phenol (CAS 108-95-2), 4-methylphenol (p-cresol, CAS 106-44-5), 2-methylphenol (o-cresol, CAS 95-48-7), 3-methylphenol (m-cresol, CAS 108-39-4), 2-methoxyphenol (guaiacol, CAS 90-05-1), 2,4-dimethylphenol (CAS 105-67-9), 4-ethyl phenol (CAS 123-07-9), 2-methoxy-4-methyl phenol (creosol, CAS 93-51-6), indole (CAS 120-72-9), 2-methoxy-4-(prop-1-en-1-yl)phenol (isoeugenol, CAS 97-54-1), 2,6-dimethoxy phenol (CAS 91-10-1), 2,2-dimethoxy propane (CAS 77-76-9), benzene (CAS 71-43-2) and naphthalene (CAS 91-20-3). A six point calibration covered the concentration range of ~5 to ~150 μg/mL. Dodecane (CAS 112-40-3) was used as an internal standard.

Each bio-oil solution was analyzed in triplicate so that the relative standard deviation (RSD) could be determined, with regular blank runs (solvent only) performed to avoid carryover of species from one sample to the next. The RSD derived from the calibration and sample data was ≤5%. The lower limit of quantification (LLQ) for each compound in the calibration is presented in Tables S3.1–3.4 in S3 File. The LLQ is defined as the point where the calibration became unreliable, i.e. where the RSD exceeded 20%.

Note: The compounds removed from the bio-oil samples during rotary evaporation are referred to as 'volatile bio-oil' in the tables. In almost all cases the 'volatile bio-oil' yields are below the LLQ for the calibrated compounds (17 compounds). The reason for these low concentrations is mainly due to the amount of solvent required to recover the bio-oil from the traps (~1 L). If each calibrated compound was present in the sample at the LLQ it would introduce a total bias of ~2.0 wt% to the volatile bio-oil yield relative to the feedstock (daf). However, this '~2 wt%' bias is probably a gross underestimate due to the low concentration of the solutions (~3 mg/mL) which is further exacerbated as less than half the peaks observed in the bio-oil GC chromatograms are calibrated. The concentration of the bio-oil solutions would have to be increased by an order of magnitude to obtain more reliable GCMS data.

### Elemental Analysis of the bio-oils and char (for C, H and N)

The carbon, hydrogen and nitrogen contents of the 'dry bio-oil' and char samples were determined by combustion analysis (Exeter Analytical Model CE 440 elemental analyzer). Using 3 to 6 mg subsamples, a minimum of five (typically eight) analyses were performed on each dry bio-oil and char sample. The standard deviations associated with these analyses are reported in the results section. The bias of the instrument, based on analysis of known samples, is on the order of 1.0% relative [42].

### Permanent Gas analysis (CO, CO_2_, CH_4_ and H_2_)

Permanent gases were analyzed using two online gas analyzers, 1) a Uras 10E three channel non-dispersive infrared analyzer for CO, CO_2_ and CH_4_, and 2) a Caldos 5G continuous flow thermal conductivity detector for H_2_. The analyzers were supplied by Applied Automation/Hartmann & Braun, Bartlesville, USA. The detectors were calibrated using certified zero and span gases.

Values reported for permanent gases can only be considered as 'indicative' due to cross interference between gas species and the transient nature of the gases emitted from the batch pyrolyzed sample. In a previous gasification study using the same analyzers the results were found to be very close to those obtained from GC analysis [43]. The greatest deviation was for CO_2_, where in the worst case, the online analyzer was determined to be 10% (relative) lower than the GC measurement. Therefore the bias in the permanent gas yield determinations is estimated to be ± 10% (relative); although the bias may be higher due to the lower amount of producer gas generated in the present study compared to gasification, e.g. nitrogen accounted for >95 vol. % of the gas passing through the analyzers. The repeatability of the permanent gas yields are presented in the results section. The term 'permanent gases' refers solely to CO, CO_2_, CH_4_ and H_2_ and not to other gases formed during pyrolysis.

## Results—Feedstock Properties

Results of the ultimate, proximate, compositional and elemental ash analyses of banagrass are presented in Table 2. The values are in close agreement with those previously reported for banagrass [4, 28]. The composition of the ash is also fairly typical for 'herbaceous and agricultural grasses (HAG)' [28], although the ash content of banagrass is greater than most other types of biomass or HAG energy crops such as switchgrass. In particular, K_2_O (~27 wt%) and Cl (~12 wt%) are present in very high concentrations in the ash and there are significant amounts of MgO and CaO (~2.5 wt% each). SiO_2_ accounts for about 50 wt% of the ash.

**Table 2:** Summary of fuel analyses results for banagrass.

	Air dry	Dry	Ground*
Proximate analysis (wt%)			
Moisture	10.7	0.0	3.0
Ash	7.6	8.5	8.2
Volatiles	74.3	83.3	80.8
Fixed C	7.4	8.3	8.1
Ultimate analysis (wt% ash-free)			
Moisture	11.6	0.0	3.3
Carbon	45.2	51.1	49.5
Hydrogen	5.1	5.7	5.5
Nitrogen	0.5	0.5	0.5
Sulfur	0.1	0.1	0.1
Oxygen^#^	37.5	42.5	41.1
Chlorine	1.1	1.3	1.2
Compositional analysis (wt% dry-ash-free)			
Lignin		23.5	
Cellulose		35.5	
Hemi-cellulose		17.5	
Ash analysis (wt% dry basis, 600°C)			
SiO_2_		47.1	
Al_2_O_3_		0.8	
TiO_2_		<0.01	
Fe_2_O_3_		0.5	
CaO		2.6	
MgO		2.5	
Na_2_O		0.5	
K_2_O		27.0	
P_2_O_5_		6.1	
SO_3_		1.5	
Cl		12.1	
CO_2_		0.8	

* 'Ground' refers to the banagrass after it had been ground to a particle size of ≤200 μm.

^#^ Oxygen by difference

Repeatability estimated to be ≤0.5% (absolute)

### Results—cellulose data, reactor performance and uncertainty

Tests were first performed with cellulose to evaluate the repeatability performance of the reactor system in terms of product distribution, with bias being assessed through comparison to literature values. Only four process conditions were examined with cellulose, two bed positions (BP-1 gives the maximum residence time and BP-4 the minimum RT) and two temperatures (400 and 600°C). Table 1 presents the set of 16 residence times spanning an order of magnitude range from 1.2 s to 12.2 s (i.e. four temperatures and four bed positions) that were used to pyrolyze banagrass.

The bio-oil, char, and gas yields from cellulose pyrolyzed at the longest residence time (BP-1) are presented in Table 3 and for the shortest residence time (BP-4) in Table 4. A summary of the char yields from cellulose pyrolysis are given in Table 5. Repeat experiments were made with cellulose and banagrass to determine standard deviations as shown in Tables 3 and 6, respectively. For all the other conditions only one experiment was performed due to time considerations. The repeatability of the data, as shown in Tables 3 and 6, is considered representative of the general reactor performance.

**Table 3:** Bio-Oil, char and gas yields (daf) from cellulose pyrolysis, at the longest residence times (BP-1).

Temperature	Dry Bio-oil	Volatile Bio-oil^#^	Char*	^CO CO_2_ CH_4_ H_2_	Undetected**
°C	wt%	wt%	wt%	wt%	wt%
400	60.5	n.d.	3.2	n.d.	n.a.
400	63.1	n.d.	3.7	n.d.	n.a.
400	62.3	n.d.	4.4	n.d.	n.a.
400	64.3	n.d.	2.8	5.9	27.0
600	16.2	n.d.	0.6	50.6	32.5
600	16.9	n.d.	0.2	44.5	38.4
600	12.7	n.d.	1.3	42.8	43.1
400 mean	62.5	n.a	3.5	n.a.	n.a.
400 S.D.	1.4	n.a	0.6	n.a.	n.a.
600 mean	15.3	n.a	0.7	46.0	38.0
600 S.D.	1.8	n.a	0.4	3.3	4.3

^#^ Volatile bio-oil refers to the amount of bio-oil removed from the sample during rotary evaporation and is determined by analyzing the bio-oil solution by GCMS before drying and again after it is dried.

^ Indicative values derived from on-line gas analysis.

* The bias in the char yield is estimated to be ≤±2 wt% (absolute).

** 'Undetected' is derived as: 100%—(dry bio-oil + volatile bio-oil + char + CO, CO_2_, CH_4_ and H_2_ yields).

n.d. not determined, due to instrument unavailability; n.a. not applicable; S.D. is the standard deviation.

**Table 4:** Bio-oil, char and gas yields (daf) from cellulose pyrolysis, at the shortest residence times (BP-4).

Temperature	Dry Bio-oil	Volatile Bio-oil^#^	Char*	^CO CO_2_ CH_4_ H_2_	Undetected**
°C	wt%	wt%	wt%	wt%	wt%
400	69.5	0.1	2.6	2.4	25.4
600	55.3	<LLQ	1.8	11.8	31.1

<LLQ, less than the lower limit of quantification.

^#^ Volatile bio-oil refers to the amount of bio-oil removed from the sample during rotary evaporation and is determined by analyzing the bio-oil solution by GCMS before drying and again after it is dried.

^ Indicative values derived from on-line gas analysis.

* The bias in the char yield is estimated to be ≤±2 wt% (absolute).

** 'Undetected' is derived as: 100%—(dry bio-oil + volatile bio-oil + char + CO, CO_2_, CH_4_ and H_2_ yields).

**Table 5:** Summary of char yields (daf) from cellulose pyrolysis.

Temperature	Mean Char	Standard Deviation	Number of tests
°C	wt%	wt%	n
400	*3*.*3*	*0*.*6*	5
600	*1*.*0*	*0*.*6*	4

The bias in the char yield is estimated to be ≤± 2 wt% (absolute).

**Table 6:** Bio-oil, char and gas yields (wt% daf) from banagrass pyrolysis at longest residence times (BP-1).

Temperature	Dry Bio-oil	Volatile Bio-oil^#^	Char_org_ *	^CO CO_2_ CH_4_ H_2_	Undetected**
°C	wt%	wt%	wt%	wt%	wt%
400	29.1	<LLQ	8.0	8.1	54.8
450	27.7	<LLQ	4.5	12.0	55.8
450	27.0	<LLQ	3.9	12.5	56.6
450	28.1	<LLQ	4.1	11.4	56.4
500	25.3	<LLQ	3.2	15.0	56.5
600	9.7	<LLQ	1.4	36.7	52.1
600	10.0	<LLQ	1.7	34.6	53.7
600	9.3	<LLQ	n.a.	33.6	n.a.
450 mean	27.6	<LLQ	4.2	12.0	56.3
450 S.D.	0.4	n.a.	0.3	0.4	0.4
600 mean	9.7	<LLQ	1.5	35.0	52.9
600 S.D.	0.3	n.a.	0.1	1.3	0.8

<LLQ, less than the lower limit of quantification.

^#^ Volatile bio-oil refers to the amount of bio-oil removed from the sample during rotary evaporation and is determined by analyzing the bio-oil solution by GCMS before drying and again after it is dried.

^ Indicative values derived from on-line gas analysis.

* The bias in the char yield is estimated to be ≤±2 wt% (absolute), values are for the daf char

** 'Undetected' is derived as: 100%—(dry bio-oil + volatile bio-oil + char + CO, CO_2_, CH_4_ and H_2_ yields).

The results presented in Table 3 characterize the pyrolysis bio-oil, char, and gas yields obtained from the reactor system at two temperatures using cellulose as feedstock at the maximum residence time, BP-1. At 400°C, the dry bio-oil and char yields were 62.5 ±1.4 wt% and 3.5 ±0.6 wt%, respectively, of the daf feedstock. Note that the uncertainty is the standard deviation of repeated experimental results and is reported on wt% absolute basis. At 600°C, the dry bio-oil and char yields were 15.3 ±1.8 wt% and 0.7 ±0.4 wt%, respectively, of the daf feedstock. Measurements at 600°C provided an estimate of gas yields of 46 ±3.3 wt%. The “Undetected” amount in Tables 3, 4 and 6 includes gases other than CO, CO_2_, CH_4_ and H_2_ and volatile material that could not be quantified by GCMS due to interference by the solvent (acetone/methanol), including pyrolysis water. This is in addition to compounds that were detected by GCMS but were not calibrated (at least half the peaks in the chromatograms were not calibrated) and poor quantification of calibrated compounds due to the low concentration of the bio-oil solutions as described in the GCMS experimental section.

**Fig 2:**
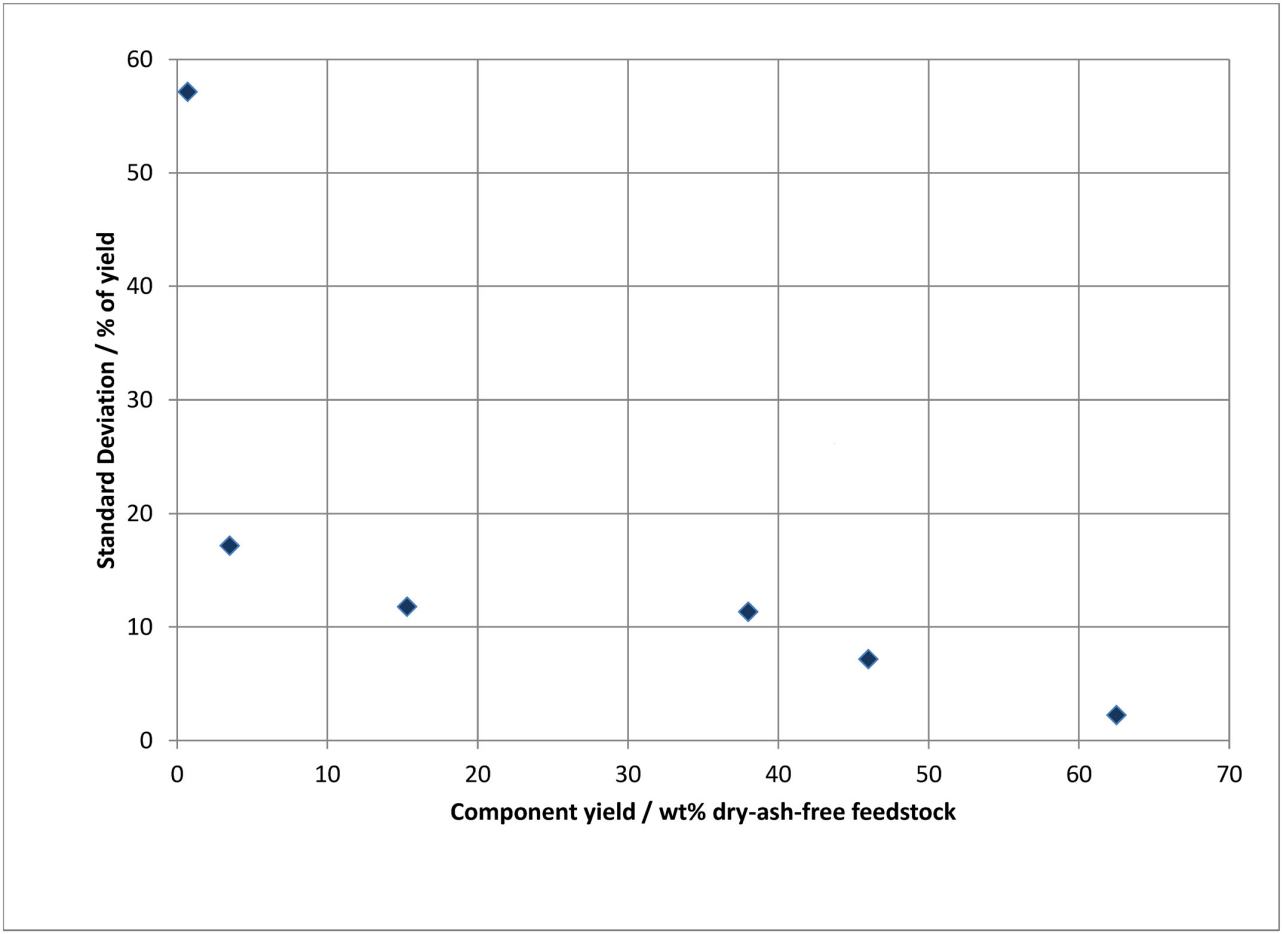
Component yields and their associated standard deviations.

*Reactor performance and uncertainty*: The standard deviations in Table 3 are presented as percentages of the measurements in Fig 2. The uncertainty follows a decreasing trend as the product yield increases. Table 6 displays the repeatability of product yields from banagrass pyrolysis. Despite the less homogeneous molecular structure of the banagrass compared to cellulose, the standard deviations for repetitive bio-oil and char results are <0.5 wt% (absolute) and for permanent gas results are <1.5 wt% (absolute). The data indicate excellent performance of the reactor system and experimental protocols.

**Fig 3:**
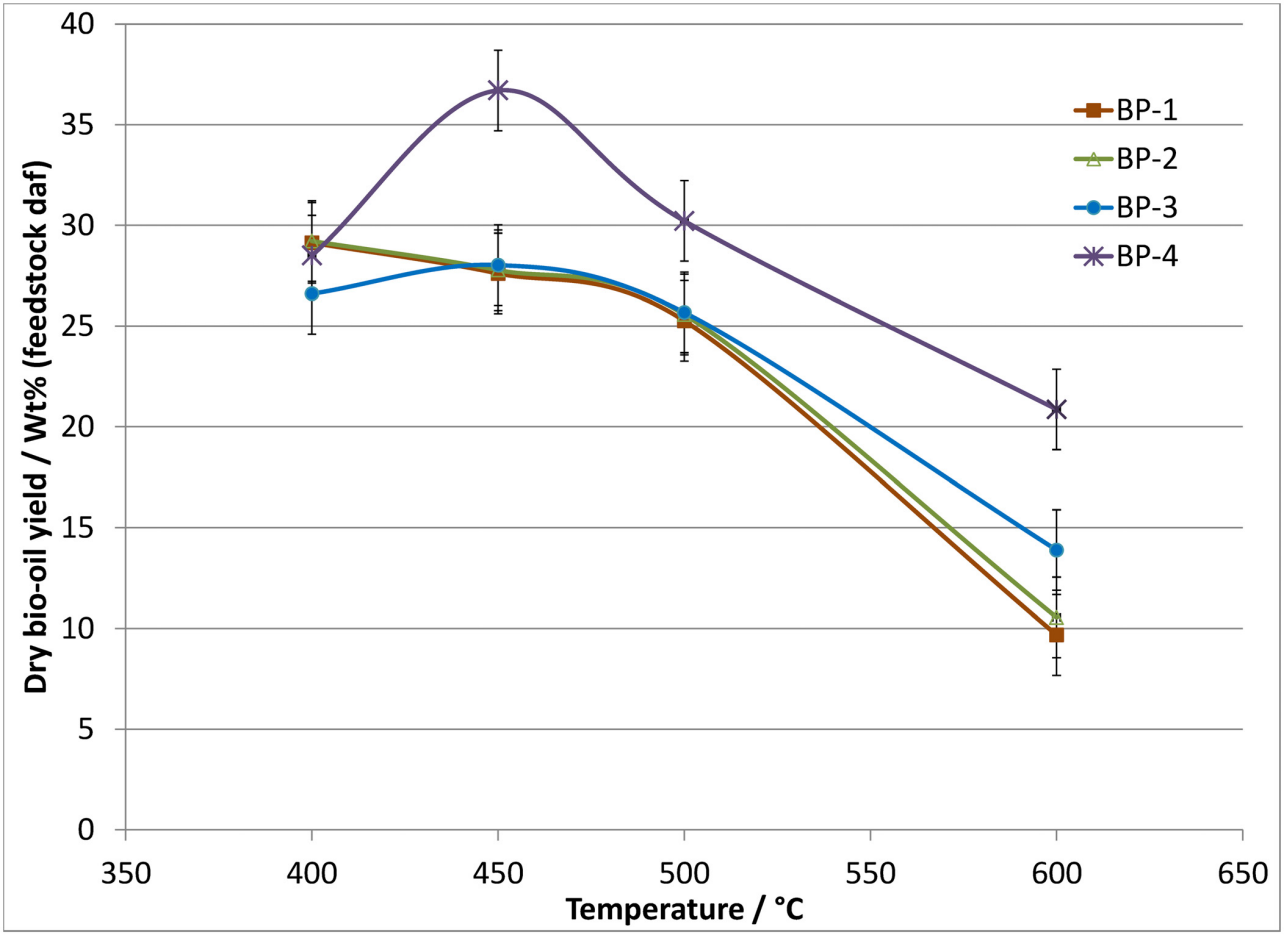
Dry bio-oil yields relative to daf feedstock as a function of temperature and vapor residence time, S.D. ≤2.0 wt% absolute.

To further assess the reactor performance and to check for systematic bias in the results, the bio-oil yields from cellulose pyrolysis are compared to those reported by Stiles et al. [21]. As shown in Table 4, the shortest residence times (BP-4) is 1.5 s at 400°C and 1.2 s at 600°C, the bio-oil yields are 69.5 wt% and 55.3 wt% of the daf feedstock, respectively. The corresponding bio-oil yields reported by Stiles for a residence time of 1.21 s are ~80 wt% and ~60 wt% at 400 and 600°C, respectively; the values were estimated from a graphical representation of their results (Fig 3, Stiles et al. [21]). The 80 wt% oil yield from cellulose pyrolysis at 400°C has also been confirmed by Fraga who used a wire-mesh reactor which greatly reduces extra-particle reactions (i.e. vapor RT <0.1 s) [6, 44]. The yields from the present study are ~10 wt% (absolute) lower than reported by Stiles at 400°C and ~5 wt% at 600°C. The possible reasons for the differences in bio-oil yield between the two studies are discussed below.

The char yields from cellulose pyrolysis at 400 and 600°C are 3.3 and 1.0 wt% daf (S.D. 0.6 wt%), respectively (Table 5). The corresponding char yields reported by Stiles at 400 and 600°C were 3 wt% (daf) and "a value too small to be determined", respectively, and both values were less than the uncertainty (±5% of daf feedstock, absolute) associated with the measurement [21]. Despite this, the char yields from Stiles show close agreement with the Table 5 values. Differences in char yields between the two studies are therefore not the cause of differences in the cellulose pyrolysis oil yields. Incomplete bio-oil recovery, cracking of the bio-oil vapors into lighter products, experimental error, or slight differences in experimental conditions are possible reasons.

The lower cellulose bio-oil yields (Table 4) compared to those reported by Stiles [21] are unlikely to be related to slight differences in residence times between the two studies; i.e. in the present study the residence time at 400°C is ~1.5 seconds and ~1.2 seconds at 600°C, while in the Stiles study the residence time at 400 and 600°C is 1.21 seconds. This is because Stiles data shows that at 400°C the bio-oil yield from cellulose was ~80 wt% over a range of residence time from 0.25 s to 2.44 s. Therefore, a more probable cause for the different bio-oil yields is a difference in the temperature regime across the bed and free-board between the two studies. In Stiles’s work, a single temperature measurement was made in the bed to control the single heating-zone furnace. In the present study two temperature measurements are made across the bed as well as four positions along the freeboard. The three furnace heating zones were set to produce as uniform a temperature profile as possible across the bed and freeboard. Therefore, in the present study the temperature in the freeboard is likely to be higher than in Stiles’s work. For example, the bio-oil yield from cellulose at 450°C reported by Stiles is ~74 wt% at 1.21 s residence time, which is similar to the bio-oil yield at 400°C in the present study (~70 wt%).

The temperature distribution across the bed and freeboard for each reaction condition is given in Tables S4.1 to S4.4in S4 File. Here it is shown that at shorter residence times the temperature distribution is less uniform due to the position of the bed relative to the three zones of the furnace. For example, when the bed support plate is in its highest position (BP-4) both the bed and freeboard are mainly being heated by the top zone of the furnace. This means it is not possible to maintain the same temperature in the freeboard as in the bed.

The other main source of bias identified for the bio-oil yield was related to deposition of bio-oil in the side-arm where pyrolysis vapors exit the freeboard enroute to the oil traps. Heavy-oil deposits were observed in the section of the side-arm that transitions from being heated to being cooled. This occurs in the ~5 cm of tubing immediately after the heated flange connection and immediately preceding the dry ice-cooled section leading to the first trap. Despite the cooling, it appears that bio-oil that deposits in this section quickly ages (recombination/polymerization reactions) into a heavy oil/tar due to the tubing still being hot. It is probable that the heavier bio-oil compounds preferentially deposit in this region due to their higher condensation temperatures. Although it is not possible to quantify the deposited bio-oil fraction, it appears to be minor compared to the total experimental mass balance, given the geometry of the arm and possible maximum amount of condensation in this area.

The potential sources of bio-oil losses due to deposition, as discussed above, would not be unique to the present study; i.e. the same types of losses would be anticipated in Stiles’s results due to the similarities of the reactor designs. It is probable, therefore, that the differences in the bio-oil yields reported here and in the Stiles study are a reflection of differences in experimental conditions and / or experimental error.

In summary of the cellulose results, the reactor system shows good repeatability with a standard deviation of ≤2.0 wt% for dry bio-oil and char yields, and ≤3.5 wt% for the gas yields. There may be a systemic bias in the data, however, that underestimates cellulose bio-oil yield by a maximum of ~10 wt% (absolute) at 400°C and ~5 wt% at 600°C. This level of bias is considered acceptable in light of the points described above and is probably a reflection of small differences in experimental conditions (temperature and residence time) and/or due to experimental errors.

### Results—Banagrass pyrolysis, bio-oil, char and gas yields

**Fig 4:**
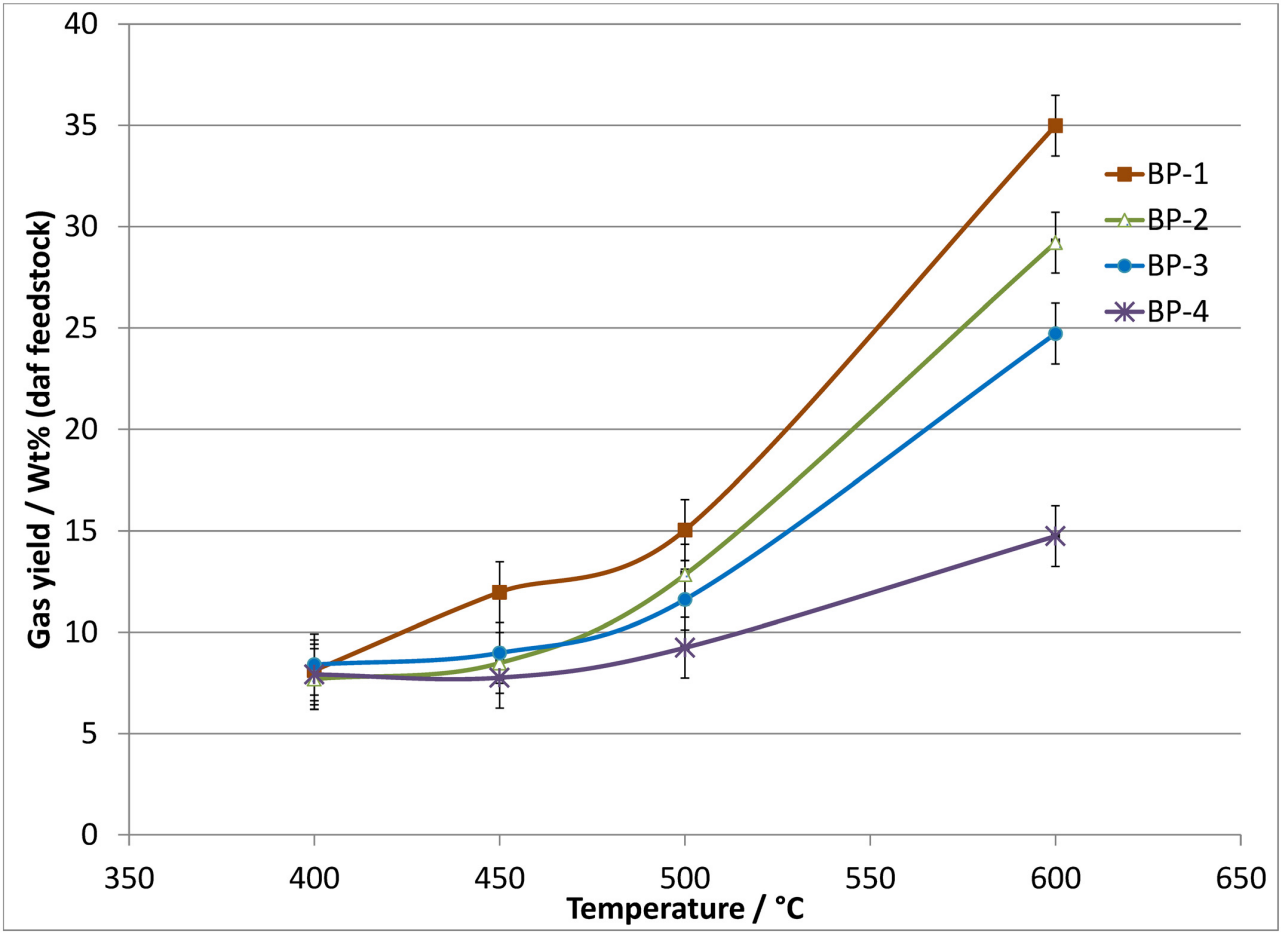
Permanent gas yields relative to daf feedstock as a function of temperature and vapor residence time, S.D ≤1.5 wt% absolute.

The bio-oil, char and gas yield data obtained from the pyrolysis of banagrass as a function of temperature at the longest residence time (see Table 6) demonstrate the experimental repeatability. Figs 3 and 4 show the dry bio-oil and permanent gas yields, respectively, as a function of temperature and vapor residence time. Char and ash yields are summarized in Table 7. The complete set of data tables can be found in Tables S5.1 to S5.3 in S5 File. A breakdown of the permanent gas data in terms of weight percent of CO, CO_2_, CH_4_ and H_2_ relative to the amount of feedstock (daf) is presented in S6 File.

**Table 7:** Summary of char and ash yields from banagrass pyrolysis.

Temperature	Mean Char_Org_	Char S.D.	Number of tests	Char_Org+Inorg_	Ash*
°C	wt% daf basis		n	wt% dry basis	
400	8.4	0.4	3	14.8	5.7
450	3.9	0.4	6	8.1	3.9
500	3.1	0.3	4	6.9	3.6
600	1.7	0.5	5	5.0	3.1

Bias is estimated at ≤±2% (absolute).

* Ash refers to the ash contained within the char, given as wt% of the feedstock on a dry basis, the S.D. of the ash yield is 1.5 wt% (absolute).

Char_Org_ refers to the organic fraction of the char relative to the daf feedstock;Char_Org+Inorg_ refers to the sum of the organic and inorganic fractions of the char relative to the dry feedstock.

The data presented in Table 6 show that at the longest residence time (BP-1, ~10–12 seconds), the maximum bio-oil yield was recovered when operating at temperatures of 400 and 450°C (28 to 29 wt% bio-oil, daf). Increasing the temperature to 500°C produced a slightly lower bio-oil yield, ~25 wt% bio-oil yield. At 600°C the bio-oil yield is significantly lower, ~10 wt% bio-oil. The permanent gas data (CO, CO_2_, CH_4_ and H_2_) shows a clear trend of increasing yield with increasing temperature. Approximately 8 wt% of the daf banagrass is converted to CO, CO_2_, CH_4_ and H_2_ at 400°C, increasing to ~12 wt% at 450°C, ~15 wt% at 500°C, and ~35 wt% at 600°C. A more detailed account of the gas analysis is given in S6 File. The daf Char_org_ yields also show a clear trend with lower yields at higher temperature, decreasing from ~8.0 wt% char at 400°C to ~1.7 wt% at 600°C.

The data shows that on going from 400°C to 450°C at the longest residence time there is an increase in permanent gases by ~4.0 wt% while the amount of bio-oil is roughly constant (~28–29 wt%), and the char yield decreases by ~4.5 wt%. This implies that the additional volatile material released from the biomass at 450°C than at 400°C is not recovered as bio-oil, instead it increases the permanent gas yield.

The trends in the bio-oil, char and gas yields in response to temperature changes at BP-2 (~6–8 s) and BP-3 (~3.5–4.5 s), respectively, are virtually identical to those outlined above for BP-1. The actual dry bio-oil and gas yields at each temperature are also similar across BP-1, BP-2 and BP-3 (i.e. they are within experimental error), see Figs 3 and 4. The similarity in bio-oil and gas yields at a particular temperature across BP-1, BP-2 and BP-3 shows that increasing the vapor residence time from ~4 to ~11 seconds results in no additional cracking of the bio-oil compounds into permanent gases.

As shown in Fig 3, when operating at 400°C, the bio-oil yield at the shortest residence time (BP-4, 1.5 s) is within experimental error of the values obtained at longer residence times (BP-3 to BP-1, 4.6 to12.2 s). This implies that there is no significant increase of bio-oil vapor cracking at residence time longer than ~1.5 s when operating at 400°C. However, at 450°C there is a significant increase in the bio-oil yield at the shortest residence times, with ~37 wt% at BP-4 (1.4 s) compared to ~28 wt% bio-oil at longer times (BP-3, BP-2 and BP-1, 4.2 to 11.3 s). A small increase in the bio-oil yield is also observed at 500°C due to a shorter residence time with a bio-oil yield of ~30 wt% at BP-4 (1.3 s) compared to ~25 wt% at BP-3, BP-2 and BP-1 (4.0 to 10.6 s). At 600°C the effect of increased residence time is more significant with bio-oil yield of ~21 wt% at BP-4 (1.2 s), decreasing to ~14 wt% at BP-3 (3.5 s) and ~10 wt% at BP-2 and BP-1 (6.4 and 9.4 s).

The permanent gas data correlates well with the trends observed for the bio-oil yields, with more gas formed at higher temperature and longer residence times. At 600°C and BP-1 the highest gas yield is observed, ~35 wt%, dropping slightly at BP-2 and BP-3 with the lowest gas yield at 600°C recorded at the shortest residence time (BP-4, ~15 wt%). When operating at 500°C the gas yield at BP-1 is ~15 wt% which decreases to ~12–13 wt% at shorter residence times (BP-2 and BP-3) and to ~9 wt% at the shortest residence time (BP-4). The gas yields at 450°C are similar to those at 500°C ranging from ~12 wt% at the longest residence time (BP-1) to ~8 wt% at the shortest residence time (BP-4). The gas yields at 400°C appear to be independent of residence time with all four residence times giving a value of ~8 wt%. The gas results support the conclusion that at 400°C all the cracking of bio-oil vapors to permanent gases has occurred within the first 1.5 s and extending the residence time up to 12 s results in no increase in the permanent gas yield.

The char yields are not influenced by changes in the residence time of the vapors and are solely a function of the bed temperature and solids residence time in the bed, which is 15 minutes in all cases. The results show that the char_org_ yield (daf) decreased with increasing temperature, from ~8.4 wt% at 400°C to ~1.7 wt% at 600°C relative to the daf feedstock (Table 7).

The ash recovered from the char after pyrolysis of banagrass is ~3–4 wt% relative to the dry feedstock independent of temperature (Table 7). The ash content of the feedstock is 8.2 wt% (dry basis, Table 2). Therefore it appears that more than half the ash originally present in the banagrass is unrecovered, partitioning either to the bed or into the bio-oil. It is unlikely that ash species would be lost due to volatilization at the temperatures used in these experiments [45].

The trends in product distributions due to pyrolysis temperature described above are in agreement with the general observations reported in the literature [6, 7, 46]. The results also show that a significant proportion of the products (~55–60 wt%) were not quantified as they were not detected by GCMS or by the permanent gas analyzers as described earlier (labeled 'undetected' in Table 6 and Tables S3.1 to S3.3 in S3 File). The amount of undetected material appears to be largely independent of temperature and residence time. The amount of undetected material for cellulose was ~40 wt%. The lower amount of undetected material for cellulose than banagrass indicates that the bio-oil vapors from banagrass have undergone more extensive cracking than the cellulose vapors. This is also confirmed by the GCMS results. It is likely that this additional cracking occurs as the vapors exit the particles (intra-particle) and may relate to the high ash content of the banagrass. Additional discussion of these points is presented below.

In summary of the banagrass pyrolysis product yields, the highest bio-oil yield (~37 wt%) was recovered when operating at the shortest residence time (BP-4, ~1.4 s) and a temperature of 450°C. These conditions also produced the least permanent gases (CO, CO_2_, CH_4_ and H_2_ totaled ~8 wt% of daf fuel). Extending the volatiles residence time in the freeboard from BP-4 to BP-3 (from ~1.4 s to 4.2 s) resulted in a significantly lower bio-oil yield (~28 wt%) but only a negligibly higher gas yield (~9 wt%), at 450°C. This implies that the additional cracking of the bio-oil vapors that occurs at longer residence time when operating at 450°C does not create more permanent gases but instead forms compounds that fall in the window between methanol and CO_2_ that could not be analyzed ('undetected' in Table 6).

The results obtained from the shortest residence time show that bio-oil yield increases from ~28 wt% at 400°C to ~37 wt% at 450°C while the gas yield is ~8.0 wt% in both cases, and the corresponding char yields decrease from 8.4 wt% at 400°C to ~4.0 wt% at 450°C. These results indicate that some of the bio-oil may have been lost when operating at 400°C as the 10 wt% increase in bio-oil yield by increasing the temperature from 400 to 450°C is not matched by a 10 wt% drop in the char yield as would be expected. This discrepancy is thought to reflect experimental error and indicates that more bio-oil may have been lost to deposition in the top of the reactor when working at 400°C than at 450°C. From comparison to other types of biomass in literature [21, 44], it is probable that the 400°C bio-oil yield should be somewhat closer to the 450°C results. This 'missing' bio-oil (~5 wt%) at 400°C may partly explain the 10 wt% lower bio-oil yield from cellulose in this studied compared to that reported by Stiles [21] or Fraga [6, 44].

A breakdown of the GCMS results for the bio-oil samples is provided in Tables S3.1 to S3.4 in S3 File along with a brief discussion of the findings. It was found that no single compound is present in any of the bio-oil samples in a significant concentration, instead there are >40 peaks all at low concentration (<0.5 wt% relative to the amount of daf feedstock). The GCMS results provide limited information, partly due to the bio-oil solutions being too dilute, therefore they will not be discussed further.

### Results—Banagrass pyrolysis, CHN of the dried bio-oils

**Fig 5:**
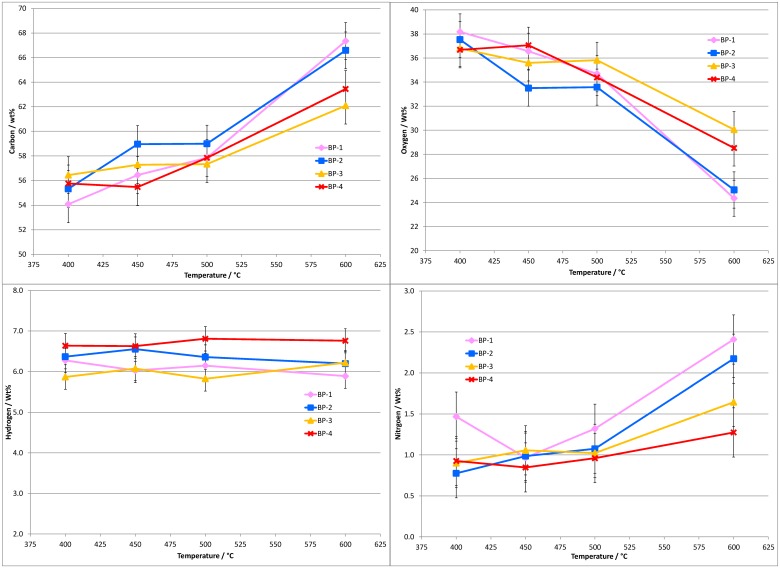
Elemental analysis (C, H, N, and O by difference) results for the dry bio-oil samples from banagrass pyrolysis as a function of temperature and vapor residence time (bed position, BP). Results are presented as wt% of the bio-oil. The standard deviation of the C and O results is ≤1.5 wt% (absolute) and for H and N ≤0.3 wt% (absolute).

C, H and N contents of the bio-oils recovered at different temperatures and vapor residence times were directly measured to provide an indication of fuel quality. O was determined by difference. Fig 5 displays the elemental analysis results for the dry bio-oils in terms of weight percent of C, H, N and O in the bio-oil.

In general, the effect of increasing residence time on the C, H, N and O contents of the bio-oil is minor compared to the influence of temperature. At 400°C the amount of carbon in the bio-oil is ~54 wt% at the longest residence time (BP-1), ~55 wt% at the 2nd longest RT (BP-2), and ~56 wt% at the shorter residence times (BP-3 and BP-4). The standard deviations of the C and O results are ≤1.5 wt% which indicates the carbon values at 400°C are within experimental error of each other. The amount of oxygen in the bio-oils also appears to be largely independent of residence time at 400°C with ~38 wt% at the longest residence time and ~36.5 wt% at the shortest residence time; i.e. these values are within experimental error of one another, but some dependence on residence time is observed at 600°C, implying that greater deoxygenation occurs at higher temperatures and residence times.

Carbon in the bio-oils produced at 450°C ranges from 55.5 to 59.0 wt% and is comparable to the data at 400°C. Similarity to 400°C data is also displayed by O, N, and H results. No clear trend due to differing residence times is displayed for any of the elements in bio-oils formed at 450°C. Differences can be attributed to experimental error.

At 500°C there is a minor increase in the amount of carbon in the bio-oil produced at the shortest residence time (BP-4) compared to 400 or 450°C (~58 wt% at 500°C, and ~55.5 to 56 wt% at 400 and 450°C). Increasing the residence time at 500°C appears to have little effect on the carbon contents of the bio-oils with all the results falling between 57.5 to 59.0 wt%.

At 600°C there is a clear increase in the carbon contents of the bio-oils compared to those produced at lower temperatures. The bio-oils recovered at the shorter residence times contain 62.0 to 63.5 wt% carbon, while at longer residence times this increases to between 66.5 to 67.5 wt% carbon. In all cases, the differences are larger than the experimental error.

Due largely to its measurement by difference, the oxygen contents of the bio-oils show opposing trends to the carbon results described above. The effect of residence time on the oxygen contents of the bio-oils is less significant than temperature and bio-oils contain markedly less oxygen at higher temperature.

The hydrogen contents of the bio-oils are independent of temperature or residence time. There is marginal evidence to suggest that the bio-oils produced at the shortest residence time contain slightly more hydrogen than at the longer residence times, at all temperatures.

For nitrogen the values are fairly similar across 400 to 500°C and at all residence times, expect for one point (400°C, BP-1) which appears high compared to the rest of the data. This is possibly an anomalous result. At 600°C the amount of nitrogen increases compared to the lower temperature bio-oils and differences due to residence time are greater.

**Fig 6:**
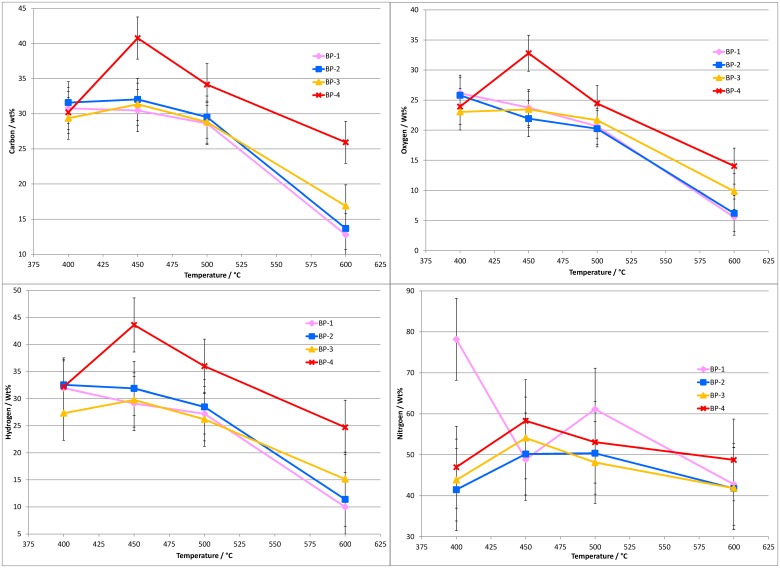
Elemental conversion efficiency (C, H, N and O by difference) results for the dry bio-oil samples from banagrass pyrolysis as a function of temperature and vapor residence time (bed position, BP). Results are presented as wt% of the element in the Feedstock (daf). The standard deviation for the C and O results is ≤3.0 wt%, for H ≤5.0 wt% and for N ≤10.0 wt% (absolute).

In summary of the elemental analysis of the dried bio-oils, the influence of residence time on the amounts of carbon and oxygen in the bio-oils appears to be minor at temperatures below 600°C. Increasing the temperature has a greater effect, the carbon contents of the bio-oils increases from ~55–56 wt% at 400°C to ~65 wt% at 600°C. For oxygen, the amount decreases from ~37–38 wt% at 400°C to ~25–30 wt% at 600°C. However, working at a higher temperature results in a significantly lower bio-oil yield (Fig 3). To account for the differing bio-oil yields at each condition, the C, H, N and O results are also presented in terms of wt% of the element relative to the total amount of the element in the feedstock (daf), Fig 6.

Fig 6 shows that the amount of carbon in the bio-oil relative to the amount of carbon in the daf banagrass is approximately constant over all four residence times when operating at 400°C (between 29 and 32 wt% C). At 450°C the amount of carbon in the bio-oils is approximately the same as at 400°C for the longer residence time bio-oils (BP-1 to BP-3), only the shortest residence time (BP-4) resulted in more carbon in the bio-oil, ~41 wt% C.

At 500°C the amount of carbon in the bio-oil at the longer residence times (BP-1 to BP-3) are within experimental error of the values at 400 and 450°C, i.e. ~29 wt% C. For the shortest residence time (BP-4) at 500°C the amount of carbon in the bio-oil is ~34 wt%. At 600°C the amount of C in the bio-oils is ~13 to 17 wt% for BP-1 to BP-3 and ~26 wt% C at BP-4, which is a significant decrease when compared to the 500°C results.

The oxygen contents of the bio-oils, when presented relative to the amount of oxygen in the daf feedstock, show the exact same trends as described above for carbon. At 400°C the amount of oxygen is between 23 and 26 wt% over all four residence times. At 450°C the amount of oxygen in the bio-oils is ~22–24 wt% for BP-1 to BP-3 and ~33 wt% for BP-4. At 500°C there is ~20 to 22 wt% oxygen in the bio-oils recovered at BP-1 to BP-3, and ~24 wt% O at BP-4. At 600°C the amount of oxygen in the BP-1 to BP-3 bio-oils is ~6 to 10 wt%, and ~14 wt% at BP-4.

The hydrogen results also show the same trends as for carbon and oxygen. At 400°C the amount of hydrogen in the bio-oils appears independent of residence time, ~27 to 32 wt% H. At 450°C there is ~29–32 wt% H in the BP-1 to BP-3 bio-oils and ~44 wt% for BP-4. At 500°C the amount of H appears to decrease slightly for BP-1 to BP-3 bio-oils compared to the 400 and 450°C results with ~26–29 wt% H, for BP-4 there is ~36 wt% H. At 600°C there is a significant decrease in the amount of hydrogen in the bio-oils than at lower temperatures with ~10–15 wt% H for BP-1 to BP-3 and ~25 wt% H for BP-4.

The results for nitrogen are less clear due to the lower absolute amounts of nitrogen present in the feedstock which inevitably results in more significant errors. Overall, the trends in the nitrogen results are the same as for carbon, oxygen and hydrogen with the notable exception of the longest residence time (BP-1) data set which appears to contain some anomalous results. The amount of nitrogen in the bio-oils relative to the nitrogen in the feedstock is ~40–60 wt% (typically ~50 wt%) over all the conditions studied. Considering the repeatability of the data (S.D is 10 wt% absolute), all the values can be considered to be within experimental error.

Elemental analysis was also performed on the char samples. Table 8 present the C, H, N and O results as weight percent of the char (daf). Table 9 present the same data in terms of weight percent of the element in the feedstock (daf).

**Table 8:** Elemental analysis results for the chars (daf), given as wt% of the char (daf).

Temperature	C	H	N	O
°C	wt%	wt%	wt%	wt%
400	61.0	2.8	1.0	35.2
450	65.2	2.6	1.1	31.1
500	65.4	2.3	0.9	31.4
600	75.4	2.4	1.1	21.0

RSD is estimated to be <20%.

**Table 9:** Elemental analysis results for the chars (daf), given as wt% of the element in the feedstock (daf).

Temperature	C	H	N	O
°C	wt%	wt%	wt%	wt%
400	10.0	4.1	14.7	7.0
450	5.0	1.8	7.9	2.9
500	4.0	1.2	5.1	2.3
600	2.5	0.7	3.4	0.8

RSD is estimated to be <20%.

The carbon contents of the chars, relative to the mass of char_org_ (daf) ranges from ~60–65 wt% in the temperature range from 400 to 500°C to ~75 wt% at 600°C (Table 8). The oxygen contents of the chars are between 30 and 35 wt% at 400 to 500°C, decreasing to ~20 wt% at 600°C. The hydrogen contents of the chars are between 2 and 3 wt% over all four temperatures and nitrogen is ~1 wt% in all cases. The repeatability of the char elemental analyses is fairly poor due to the small sample size and difficulty of recovering clean char samples, the standard deviation is up to 20% (relative).

Presenting the elemental analysis results for the chars relative to the amount of the element in feedstock (daf) reveals clear trends (Table 9). The amount of carbon in the char decreases with increasing temperature, from ~10 wt% at 400°C to ~2.5 wt% at 600°C. For oxygen the maximum is ~7 wt% at 400°C decreasing to ~1 wt% at 600°C. Hydrogen decreases from ~4 wt% at 400°C to ~1 wt% at 600°C, and nitrogen decreases from ~15 wt% at 400°C to ~3 wt% at 600°C.

The results from elemental analysis of the chars are in general agreement with the trends reported in literature.[31] The chars contain low amounts of carbon and hydrogen relative to the amounts present in the feedstock (≤10 wt% for carbon and ≤5 wt% for hydrogen).

### Results—Comparison of cellulose and banagrass product yields

To gain a better understanding of the banagrass pyrolysis results our findings are compared with similar studies of other solid fuels. The reactor used in this study is based on a design by Stiles et al. [21] who examined two coals, one biomass (silver birch—a hardwood) and municipal solid waste (MSW). Therefore, it is relevant to compare our findings with those reported by Stiles (see Tables 10 and 11). Bio-oil and char yields from other biomass species converted in fluidized beds under similar conditions are also included in Tables 10 and 11, respectively [22, 23, 26, 46, 47]. Ash, AAEM, lignin and volatiles contents (wt% dry basis) are also listed in Table 10. In the present study the dry bio-oil yields exclude pyrolysis water as it was removed along with the solvent during rotary evaporation (true also for Stiles’s results). The other literature sources either report a bio-oil yield inclusive of pyrolysis water (whole bio-oil) or the organic fraction of the bio-oil and pyrolysis water separately. The 'dry bio-oil' yields in Table 10 are loosely comparable to the 'organic bio-oil' yields.

**Table 10:** Bio-oil yields from the fast pyrolysis of various biomass species presented as wt% relative to the feedstock (daf), feedstock particle size <1000 μm.

	Banagrass	Silver Birch	Willow SRC	Pine saw dust*	Switchgrass	Miscanthus	Wheat straw	Barley straw	Rapeseed Straw*	Rice straw	Sugarcane Bagasse	Sugarcane Bagasse
Temperature	Dry Bio-oil	Dry Bio-oil	Organic Bio-oil	Organic Bio-oil	Organic Bio-oil	Organic Bio-oil	Organic Bio-oil	Organic Bio-oil	Organic Bio-oil	Whole bio-oil	Whole bio-oil	Whole bio-oil
°C	wt% daf											
~400	28	56	-	-	-	-	-	-	-	44	53	48
~450	37	56	-	-	-	-	-	-	-	-	-	-
~500	30	52	43–47 (10–16)	62 (11)	49–53 (11–13)	43 (6)	23 (14)	38 (17)	48 (13)	28	50	54
RT (s)	1.3–1.5	1.2	<1.5	0.5–2.0	<1.5	<1.5	<1.5	0.6–0.7	0.5–2.0	1.2	2.0	<0.1
Ash (d.b. wt%)	8.2	0.3	3.0	0.1	2.6–5.7	4.5	4.9	5.8	6.1	9.2	6.2	1.6
AAEM (d.b. wt%)	~2.8	-	~1.9	<0.05	~0.7	~1.5	~1.2	~2.4	~2.8	~2.0	-	-
K (d.b. wt%)	2.30	-	0.59	~0.03	0.09	1.20	0.57	~2.0	~1.3	1.61	-	-
Na (d.b. wt%)	0.04	-	0.01		0.02	0.00	0.01			0.03	-	-
Mg (d.b. wt%)	0.21	-	0.16	~0.01	0.06	0.15	0.07	~0.4	~1.5	0.09	-	-
Ca (d.b. wt%)	0.22	-	1.15		0.50	0.18	0.51			0.23	-	-
Volatiles (d.b. wt%)	83	-	81	84	83	76	80	74	78	-	73	-
Lignin (d.b. wt%)	24.4	18.4	19.0–20.0	~29	6.1–12.0	12.5–14.9	7.5–23.4	-	-	19.3	-	-
Reference	Present	Stiles[21]	Greenhalf[23]	Oasmaa[22]	Greenhalf[23], Boateng[26]	Greenhalf[23]	Greenhalf[23]	Oasmaa[22]	Oasmaa[22]	Eom[47]	Xu[46]	Fraga[44]

Values in parentheses show the pyrolysis water yield.

*Particle size 3–5 mm.

**Table 11:** Char_org_ yields (estimated to a daf basis) from the fast pyrolysis of various biomass species presented as wt% relative to the feedstock (daf), feedstock particle size <1000 μm.

Temp	Banagrass	Silver Birch	Willow SRC	Pine saw dust*	Switchgrass	Miscanthus	Wheat straw	Rice straw	Sugarcane Bagasse	Sugarcane Bagasse
°C	wt% daf									
~400	8	17	-	-	-	-	-	18	26	11
~450	4	10	-	-	-	-	-	-	-	-
~500	3	6	17–18	12	10–14	26	23	11	20	6
Reference	Present	Stiles[21]	Greenhalf[23]	Oasmaa[22]	Greenhalf[23], Boateng[26]	Greenhalf[23]	Greenhalf[23]	Eom[47]	Xu[46]	Fraga[44]

*Particle size 3–5 mm.

Table 10 shows that woody biomass typically produces the greatest bio-oil yield. The yields from sugarcane bagasse and switchgrass fall within the lower end of the range of wood values, with miscanthus giving a significantly lower yield. The lowest yields are from the straws except rapeseed straw which is similar to bagasse. Banagrass gives a similar bio-oil yield as barley straw, which is significantly greater than the yields from wheat straw and rice straw.

If the ash, AAEM, lignin and volatiles contents are also considered (Table 10) more detailed conclusions can be drawn. Banagrass and rapeseed straw have similar volatiles (~80 wt%) and AAEM (~2.8 wt%) contents but banagrass produces much less bio-oil. The reason for this appears to be related to the differing amounts of Na + K verses Ca + Mg for banagrass and rapeseed straw. Rapeseed contains ~1.5 wt% of Ca + Mg and ~1.3 wt% of K + Na, whereas banagrass contains 2.3 wt% K and low amounts of Ca + Mg (~0.4 wt%). This indicates that K has a greater effect on reducing the bio-oil yield than Ca and Mg. Barley straw produces a similar amount of bio-oil as banagrass and it contains similar AAEM contents where K and Na are the dominant species (~2.0 wt%) and Ca + Mg account for ~0.4 wt%. These observations appear to support the assertion that K and Na play a more significant role in suppressing the bio-oil yield than Ca and Mg. The role of lignin is less clear, partly due to a lack of accurate data (Table 10). Nonetheless, it appears that samples with low amounts of AAEM and high amounts of lignin produce the greatest bio-oil yields (i.e. woods).

It is unclear why wheat straw produces so little bio-oil and so much char and gas, according to Greenhalf et al. [23] the reason is the high concentration of K. The ash content of wheat straw is fairly high (~5 wt%), but the AAEM content is medium (~1.2 wt%) when compared to the other samples in Table 10. The amounts of K + Na in wheat straw is ~0.6 wt% which is similar to willow and less than miscanthus (1.2 wt% K + Na) or banagrass (K is 2.3 wt%). It is unlikely therefore that K on its own is responsible for low bio-oil yield from wheat straw. It is possible that the low bio-oil yield from wheat straw is related to its lignin content. However, the lignin content of wheat straws appears to be highly variable with literature values ranging from 7.5 to 23.4 wt% (Table 10).

Table 11 shows that the char yield from banagrass is significantly lower than any of the other materials and that the results from Stiles and Fraga are similar. This may be related, in part, to the small particle size used in this study (<200 μm) and by Stiles and Fraga (100–150 μm) compared to the other results shown in Table 11 (typical partial size 500–1000 μm). Nonetheless it is clear that banagrass does not produce significant amounts of char under fast pyrolysis conditions. A similar low char yield (~14 wt% dry basis) was reported for elephant grass from a 200 kg/h fast pyrolysis pilot plant (feedstock particle size 3 mm, ~5 wt% ash).[31]

Stiles et al. [21] reported bio-oil yields of ~56 wt% for silver birch at 450°C and residence time of 1.21 s, with ~59 wt% bio-oil at 0.25 s RT and extrapolated back to estimate the bio-oil yield at zero residence time (~60 wt%). The corresponding results for cellulose (450°C) were ~74 wt% at 1.21 s residence time and ~77 wt% for zero residence time. In both examples, the extent of bio-oil vapor cracking in the first 1.2 s is 3–4 wt% (absolute). Fraga et al. [44] reported bio-oil and char yields for silver birch from a wire-mesh reactor where the volatile residence time was effectively zero seconds (<0.1s). Fraga's silver birch results are similar to that of Stiles, where the bio-oil yield (daf basis) at zero RT is ~56 wt% at 400°C and ~58 wt% at 500°C; char yields were ~11 wt% at 400°C and ~4 wt% at 500°C. Admittedly this is not an ideal comparison due to the differences in stability of the bio-oil vapors from cellulose, silver birch and banagrass; however, if we assume the same extent of bio-oil vapor cracking for banagrass as for silver birch and cellulose we can estimate a banagrass 'dry bio-oil' yield of ~42 wt% at 450°C with zero residence time.

Pyrolysis water typically accounts for 10–20 wt% of products from biomass on a daf basis, as shown in Table 10. Biomass species with high ash contents and/or high concentrations of AAEM typically produce less pyrolysis water and more light/volatile compounds than species with low amounts of AAEM according to Fahmi et al. [24], although this is not apparent from the data in Table 10. Based on Fahmi’s results [24] and the data in Table 10, we can estimate the amount of pyrolysis water from banagrass to be ~15 wt% relative to the daf feedstock. The amount of useful volatile material from banagrass pyrolysis can be estimated by subtracting the amounts of CO_2_ and pyrolysis water from the total volatiles yield. Total volatiles from banagrass at 450°C and 1.4 s RT account for ~95 wt% of the daf feedstock, CO_2_ accounts for ~2.5 wt% and pyrolysis water is estimated to be ~15 wt%. By difference, ~77 wt% of the daf banagrass is converted into useful products, i.e. flammable/reactive gases, volatiles and bio-oil.

Comparing the present data with Stiles’s data [21] indicates that the conversion of daf feedstock to oil at the ideal conditions follows a trend of decreasing yield; cellulose > woody biomass > grasses > straws (and banagrass) > MSW > coals. The temperatures which produce the greatest oil yields from the aforementioned substrates is ~400°C for cellulose, 400–450°C for straws, ~450°C for banagrass, 450–500°C for woods and other grasses, ~500°C for MWS and >550°C for coals.

The high bio-oil yield from fast pyrolysis of cellulose is well documented, ~80 wt% or greater at residence time of ~≤1 s when secondary reactions are minimized (at 400°C) [6, 21, 48]. Under these conditions, levoglucosan accounts for as much as ~75 wt% of the bio-oil. Lignocellulosic biomass produces lower yields of levoglucosan, and bio-oil in general, than would be anticipated from the cellulose content of the biomass, as has been discussed elsewhere [6, 48]. Briefly, the low yield of levoglucosan from biomass pyrolysis is related to its reactivity at temperatures above ~350°C. Levoglucosan decomposes quickly as it interacts with the pyrolyzing solid mass of the biomass particle. More specifically, there is evidence that during biomass pyrolysis the cellulose and hemicellulose components start to decompose/react at lower temperatures than the lignin. This leads to reactions between the primary bio-oil vapors as they come into contact with remnants of solid lignin and ash species as they escape the particle. These reactions result in a low yield of levoglucosan, typically less than ~3 wt% of the bio-oil.

The amount of levoglucosan was not quantified in the present study, however it was detected during the GCMS analysis of the bio-oils. For the cellulose bio-oils, levoglucosan is the major component of the bio-oils at all temperatures and residence times, with higher yields at lower temperatures and shorter residence times, which is consistent with findings from other reactor configurations [6, 48]. For the banagrass bio-oils, levoglucosan was present at a very low concentration having a similar peak area as isoeugenol (the compound typically present in the highest concentration in the bio-oils), indicating that the concentration of levoglucosan is less than ~1 wt% relative to the amount of feedstock (daf).

In the present study, the bio-oil yield from cellulose pyrolysis at 400°C and the shortest residence time (BP-4, 1.5 s) is ~69.5 wt%, falling to 62.5 wt% at the longest residence time (BP-1, 12.2 s). The corresponding bio-oil yields from banagrass are ~28–29 wt% at 400°C, across all four residence times. This shows that the bio-oil yield from cellulose is reduced as the vapor residence time increases, whereas the bio-oil yield for banagrass was much lower and remained unchanged when increasing the residence time. This is further evidence that during banagrass pyrolysis almost all of the levoglucosan has decomposed within 1.5 seconds (at 400°C). Furthermore, the amount of bio-oil vapors entering the freeboard (zero residence time) from banagrass is unlikely to be much greater than the yield after 1.5 s residence time, based on Stiles's [21] and Fraga's [6, 44] findings as discussed earlier. Therefore, the lower bio-oil yield from banagrass, compared to silver birch and most of the other biomass materials listed in Table 10, appears to be caused by intra-particle cracking of primary bio-oil vapors as they exit the particles.

As mentioned earlier, the high ash content of banagrass (8.2 wt% ash, db, Table 2) and the high concentrations of AAEM species (K, Mg and Ca, Tables 2 and 10), in particular K, appears to be responsible for the relatively low bio-oil and char yields from banagrass. A number of researchers have shown a link between concentrations of AAEM in biomass and bio-oil and char yields and properties [9, 11, 22–24, 27, 47].

## Conclusions

In this study the fast pyrolysis behavior of banagrass was examined in terms of the influence of temperature and vapor residence time on product distributions and elemental composition of the products.

The maximum yield of 'dry bio-oil' (~37 wt%) was obtained when working at 450°C and the shortest residence time (RT 1.4 s). The corresponding char_org_ and gas yields are ~4 wt% and ~8 wt% respectively. The carbon contents of the 450°C bio-oil (RT 1.4 s) is ~56 wt%, oxygen accounts for ~37.5 wt%, hydrogen ~6.5 wt% and nitrogen ~1 wt%. The absolute amount of carbon in the 450°C bio-oil (RT 1.4 s) is ~40 wt%, relative to the element in the feedstock (daf basis), oxygen ~33 wt%, hydrogen ~44 wt% and nitrogen ~58 wt%. On the same basis, the amount of carbon in the 450°C char is ~5 wt%, oxygen ~3 wt%, hydrogen ~2 wt% and nitrogen ~8 wt%. The composition of the gas in terms of wt% relative to the daf feedstock is ~5 wt% CO, ~2.5 wt% CO_2_, <0.5 wt% for CH_4_ and <0.1 wt% for H_2_ (see S6 File). The amount of gas produced at 450°C and 1.4s residence time is ~0.1 liters per gram of banagrass (daf basis), or ~8 wt% relative to the daf feedstock. Under these conditions (450°C and 1.4 s residence time) the maximum retention of carbon in the bio-oil is achieved and the least amount of carbon is lost as CO_2_.

Working at a residence time of less than 1.0 second should increase the bio-oil yield by a few percent, as discussed earlier, and result in slightly greater retention of carbon in the bio-oil.

If the goal of fast pyrolysis is to produce a bio-oil with the lowest oxygen and highest carbon content, the conditions to use are 600°C with a residence time longer than 1.5 seconds. However, under these conditions the bio-oil yield is significantly less than at lower temperatures and shorter residence times, ~14 wt% bio-oil at 600°C, RT 3.5 s which contains ~62 wt% carbon and ~30 wt% oxygen. These conditions also produce the greatest yields of CO, CH_4_ and H_2_, but also result in a significant loss of carbon as CO_2_ (~4 wt% relative to the daf feedstock).

The results show that the primary bio-oil vapors from banagrass pyrolysis are highly reactive, the data indicates that almost all of the cracking of bio-oil vapors occurs as the vapors exit the particles (intra-particle). This is thought to be due to the high ash content of banagrass (8.5 wt%, dry basis) and in particular due to the high concentration of alkali and alkali earth metals (totaling ~2.8 wt%, dry basis). Potassium accounts for ~2.3 wt% of the daf feedstock.

The bio-oil and char yields from fast pyrolysis of banagrass are significantly lower than from a woody biomasses or grasses such as switchgrass or miscanthus, but are similar to barley straw. The reason for the low bio-oil yield from banagrass is thought to be due to the high concentration of alkali and alkali earth metals.

## Supporting Information

S1 File Fluidized bed reactor (DOCX)

S2 File Experimental procedure (DOCX)

S3 File GCMS results (DOCX)

S4 File Temperature distributions across the bed and freeboard (DOCX)

S5 File Pyrolysis product yields (bio-oil, char and gas)—data tables (DOCX)

S6 File Breakdown of the permanent gas yields (DOCX)

S1 Fig Permanent gas data (CO, CO_2_, CH_4_ and H_2_) from the pyrolysis of banagrass as a function of temperature and vapor residence time, presented as wt% of the daf feedstock. (TIF)
