# Supplementary material for: Fast Pyrolysis Behavior of Banagrass as a Function of Temperature and Volatiles Residence Time in a Fluidized Bed Reactor
Source: PLoS One. 2015 Aug 26;10(8):e0136511. doi: 10.1371/journal.pone.0136511 (PMC4550300; doi:10.1371/journal.pone.0136511)
Supplement: S1 File — (DOCX) [file pone.0136511.s001.docx]

**Supporting Information - Fast pyrolysis behavior of banagrass as a function of temperature and volatiles residence time in a fluidized bed reactor**

**S1 File. Fluidized bed reactor**

A three zone split tube furnace is used to electrically heat the reactor (Thermcraft Ltd, USA). Each zone of the furnace can be independently controlled. Two multi-point temperature probes (Omega Engineering Inc., Stamford, CT) were used to measure the axial temperature profile of the bed and free-board. The first multi-point probe contained four thermocouples (TC); T1 is located in the bed ~5 mm above the bed support plate, T2 is ~5 mm below the top of the stationary bed, T3 is ~25 mm above the bed screen and T4 is in the freeboard. A second multi-point probe contained two thermocouples; T5 is located at the exit of the free-board into the side-arm and T6 is positioned in the top of the reactor above the heat shield. The heat shield is a 38 mm thick stainless steel disc suspended from the top end-cap and located 5 mm above the side-arm exit to prevent overheating the PTFE seals in the top of the reactor.

The position of the bed support plate / gas distributor can be adjusted to alter the volatiles residence times without changing fluidizing conditions in the bed. Four inch stainless steel sanitary fittings are used to seal the top and bottom of the reactor with Swagelok tube fittings welded into the end-caps to support the drop tube, temperature probes, and the gas distributor, see Figure 1. PTFE seals were used on these connections to allow positioning within the reactor to be altered. Water cooling is applied to the top and bottom 15 cm of the reactor via external copper coils to protect the PTFE gaskets and ferrules in the end-caps from excessive heat.

As in the Stiles design the fluidizing gas enters through the 'support tube' for the bed support assembly. The 'support tube' is blocked after it enters the reactor body, with holes drilled in the tube so the gas is forced out into the reactor body and is heated through contact with the reactor walls before passing through a gas distributor packed with ceramic chips. The bed support plate assembly acts as a gas distributor and gas pre-heater. A 25 mm wide strip of woven ceramic cord is wrapped around a recessed groove in the top section of the gas distributor body to seal the space between the assembly and the reactor wall. The bed support plate is made by stretching a piece of wire mesh screen over the top of the distributor body which creates a seal against the inner wall of the reactor body when it is inserted into the reactor. The wire mesh cloth / screen used in the reactor is made of 304 stainless steel, 230 x 230 mesh, 0.0014" wire (McMaster-Carr, USA, part number: 85385T879).
