# Supplementary material for: Fast Pyrolysis Behavior of Banagrass as a Function of Temperature and Volatiles Residence Time in a Fluidized Bed Reactor
Source: PLoS One. 2015 Aug 26;10(8):e0136511. doi: 10.1371/journal.pone.0136511 (PMC4550300; doi:10.1371/journal.pone.0136511)
Supplement: S2 File — (DOCX) [file pone.0136511.s002.docx]

**Supporting Information - Fast pyrolysis behavior of banagrass as a function of temperature and volatiles residence time in a fluidized bed reactor**

**S2 File. Experimental procedure**

The reactor was heated to the desired temperature and held at these conditions for at least 45 minutes to ensure uniform heating throughout the reactor before introducing the feedstock. The feedstock was fed into the bed from a gravity-flow hopper connected to the top of the drop-tube. Approximately 7.5 g of feedstock was used for each experiment and took 1.5 to 2.5 minutes to feed into the reactor. Fuel pyrolysis was considered complete when the elevated CO concentration in the exit gas from the process returned to <0.1 vol.% as observed with online gas analyzers. At 600 °C, pyrolysis was completed within ~4-5 minutes of starting to feed the fuel; at 400 °C it took ~8-10 minutes. The majority of the gas (>90 %) was released in a 3-4 minute time interval independent of temperature. In all cases, heating and all gas flows were stopped 15 minutes after fuel feeding was initiated. Immediately after stopping the experiment, the oil traps were removed from the reactor at the side-arm flange and the bio-oil recovered.

The first oil trap contained an inline soxhlet thimble (Whatman, UK, part number: 2800-373). Both traps were packed with stainless steel wire balls (Scotch-Brite® Stainless Steel Scrubber 84-1-4) to aid heat transfer and to provide additional cold surfaces for condensation. In addition, four small pieces of wire mesh were placed in the tubing connecting the outlet of trap-1 to the inlet of trap-2 to further aid bio-oil condensation.

Bio-oil was recovered from the traps by washing with a mixture of 80 vol. % acetone and 20 vol. % methanol (HPLC grade, Fisher Chemicals). Liquids from the soxhlet extraction of the thimble from the first oil trap and the rinse from the first oil trap were combined. The rinse from the second oil trap was analyzed separately. The amount of bio-oil recovered from the second trap was always less than 5 wt% of the total bio-oil (typically <2 wt% of the total bio-oil) indicating that all the bio-oil was being captured. Bio-oil solutions were filtered after recovery (Whatman, UK, part number: 1004090).

Samples of the bio-oil solutions (trap-1 and trap-2) were analyzed separately by GCMS. The two bio-oil solutions were stored overnight at -20 °C. A rotary evaporator operating at 55 °C with a nitrogen purge and a maximum vacuum of ~25" Hg was used to remove the solvent. Three sub-samples from each bio-oil solution were dried and the mean of these determinations is defined here as the 'dry' bio-oil yield. Repeatability of the dry bio-oil yield was assessed by repeating the experiment three times, producing bio-oil solutions from the two oil traps from each experiment, and then sub-sampling and analyzing each oil trap solution three times. In total, three experiments produced a total of eighteen dry bio-oil samples. From this, a standard deviation of ≤2 wt% (absolute) of the daf feedstock was determined. The bias in the dry bio-oil yield is discussed in the results section. A sample of the dry bio-oil was dissolved in fresh solvent and analyzed by GCMS. Comparing this dry bio-oil analysis with the analysis of bio-oil solutions before they were dried provides an estimate of the ‘volatile bio-oil’ fraction removed with the solvent during rotary evaporation. The repeatability and bias of the 'volatile bio-oil' yield is discussed in the GCMS experimental section. Rotary evaporation resulted in water being lost from the bio-oil samples, therefore determination of pyrolysis water was not attempted.

After the reactor cooled down, the drop tube assembly was removed and any char caught in the bed screen collected. The top of the reactor was then capped off and the side-arm gasket, with wire mesh screen, was installed at the exit flange. The bed was fluidized with ambient temperature air at 45 LPM for 5 minutes to elutriate the char from the bed. The char was recovered from the side-arm gasket screen and weighed. The amount of char remaining in the bed was estimated by combusting the bed material in a muffle furnace at 600 °C and recording the difference in weight. Typically, less than 10 wt% of the char remained in the bed after air recovery. When the bed was in its highest position (BP-4) the air flow rate was reduced to 15 LPM to prevent sand from being ejected with the char which typically resulted in more char (up to ~30 %) remaining in the bed. The reported char yields included char recovered by the soxhlet thimble and by filtering the bio-oil solutions. The repeatability of the char yield determinations was ~±1.5 wt% (absolute). The bias in the char yield is estimated to be ≤±2.0 wt% (absolute). Char samples were ashed in a muffle furnace at 600 °C, accordingly, char yields were corrected to a dry ash free (daf) basis. Char yields are reported for the organic fraction (Char_Org_) excluding ash, i.e. on a daf basis relative to the daf feedstock. Char yields are also reported inclusive of ash (Char_Org+Inorg_) on a dry basis relative to the dry feedstock.
