# Supplementary material for: Fast Pyrolysis Behavior of Banagrass as a Function of Temperature and Volatiles Residence Time in a Fluidized Bed Reactor
Source: PLoS One. 2015 Aug 26;10(8):e0136511. doi: 10.1371/journal.pone.0136511 (PMC4550300; doi:10.1371/journal.pone.0136511)
Supplement: S3 File — (DOCX) [file pone.0136511.s003.docx]

**Supporting Information - Fast pyrolysis behavior of banagrass as a function of temperature and volatiles residence time in a fluidized bed reactor**

**S3 File. GCMS results**

Table S3.1 to S3.4 displays a summary of the quantitative results for the Banagrass pyrolysis oils as a function of vapor residence time (bed positions BP-1 to BP-4) and temperature (400, 450, 500 and 600 °C). The values represent the amount of each compound relative to the amount of feedstock (daf basis) in weight percent. The lower limit of quantification (LLQ) is also given in the tables, which is derived from the calibration data as described in the experimental section of the manuscript. A value of zero means the compound was not detected. It is important to note that the GCMS results presented below are for the absolute yield of each compound present in the oil, which is not the same as the 'volatile oil yield' referred to in the manuscript (Tables 3, 4 and 6) which is a measure of the amount of material removed from the bio-oil samples during rotary evaporation.

In summary of the GCMS results, no single compound in the GC range was found to be present in any of the bio-oil samples in a significant concentration. Instead the chromatograms showed at least ~40 peaks are detected but all at low concentrations. All the oil samples showed a large peak due to 2, 2- dimethoxypropane, which ranged in concentration from about 5 wt% relative to the amount feedstock (daf) at 400 °C, which increased significantly for the higher temperature oils, reaching a maximum of ~80 wt% for the 600 °C oils. These results are *not* included in the tables below as it is apparent that 2, 2- dimethoxypropane is a product from reactions between the oil compounds and the solvent (mixture of acetone and methanol). It is possible that some of the 2, 2 dimethoxypropane present in the oils is produced during pyrolysis as other researcher have reported this finding.[[1](#_ENREF_1)] However, in the present study it was clear that the concentration of 2, 2 dimethoxypropane greatly increased in the samples over time, this could be clearer seen from the 3 analyses that are performed on each sample, with the first analysis always containing a lower concentration than subsequent analyses.

A brief aging study was performed and the concentration of 2, 2 dimethoxypropane in the oil solutions keep increasing over a period of at least 10 days, with the biggest increases observed within the first few hours of recovering the oils and smaller increases over extended lengths of time.

Table S3.1. Quantitative GCMS results for the Banagrass pyrolysis oils recovered at the longest residence time (BP-1) over four temperatures. Results are presented as wt% relative to feedstock (daf).

| Target Compound | *LLQ* | Temperature / °C | | | |
| --- | --- | --- | --- | --- | --- |
|  |  | 400 | 450 | 500 | 600 |
|  | *ug/mL* | wt% | wt% | wt% | wt% |
| Cyclohexane | *5* | <LLQ | 0 | 0 | 0 |
| Furfural | *15* | <LLQ | 0 | <LLQ | 0 |
| 3-methyl-2-cyclopenten-1-one | *15* | 0 | 0 | 0 | 0 |
| Phenol | *5* | 0 | <LLQ | 0 | <LLQ |
| 2-methoxy-phenol | *10* | <LLQ | 0 | <LLQ | <LLQ |
| o-Cresol | *10* | <LLQ | <LLQ | <LLQ | <LLQ |
| p-Cresol | *10* | 0 | <LLQ | <LLQ | <LLQ |
| m-Cresol | *5* | <LLQ | <LLQ | <LLQ | <LLQ |
| Creosol | *5* | <LLQ | <LLQ | <LLQ | <LLQ |
| 2,4-Dimethyl-phenol | *5* | 0 | <LLQ | <LLQ | <LLQ |
| 4-ethyl-phenol | *10* | <LLQ | <LLQ | <LLQ | <LLQ |
| 2,6-dimethoxy-phenol | *5* | <LLQ | <LLQ | <LLQ | <LLQ |
| Indole | *5* | <LLQ | <LLQ | <LLQ | <LLQ |
| Isoeugenol | *10* | <LLQ | <LLQ | <LLQ | <LLQ |
| Benzene | *10* | 0 | 0 | <LLQ | <LLQ |
| Naphthalene | *10* | <LLQ | <LLQ | <LLQ | <LLQ |
| <LLQ, less than the lower limit of quantification.  0 means the compound was not detected. | | | | | |

Table S3.2. Quantitative GCMS results for the Banagrass pyrolysis oils recovered at the second longest residence time (BP-2) over four temperatures. Results are presented as wt% relative to feedstock (daf).

| Target Compounds | LLQ | Temperature / °C | | | |
| --- | --- | --- | --- | --- | --- |
|  |  | 400 | 450 | 500 | 600 |
|  | ug/mL | wt% | wt% | wt% | wt% |
| Cyclohexane | 5 | 0 | 0 | 0 | 0 |
| Furfural | 15 | <LLQ | <LLQ | <LLQ | 0 |
| 3-methyl-2-cyclopenten-1-one | 15 | <LLQ | <LLQ | <LLQ | <LLQ |
| Phenol | 5 | 0 | 0.14 | 0.07 | 0 |
| 2-methoxy-phenol | 10 | 0.16 | 0.20 | 0.15 | 0.17 |
| o-Cresol | 10 | <LLQ | <LLQ | <LLQ | <LLQ |
| p-Cresol | 10 | 0.15 | <LLQ | 0.17 | <LLQ |
| m-Cresol | 5 | 0.09 | 0.07 | 0.09 | <LLQ |
| Creosol | 5 | 0.18 | 0.19 | 0.16 | 0.11 |
| 2,4-Dimethyl-phenol | 5 | <LLQ | 0.11 | <LLQ | 0.09 |
| 4-ethyl-phenol, | 10 | 0.20 | 0.19 | 0.20 | 0.20 |
| 2,6-dimethoxy-phenol, | 5 | 0.24 | 0.22 | 0.21 | 0.27 |
| Indole | 5 | <LLQ | <LLQ | <LLQ | <LLQ |
| Isoeugenol | 10 | 0.37 | 0.38 | 0.32 | 0.42 |
| Benzene | 10 | 0 | 0 | 0 | 0 |
| Naphthalene | 10 | <LLQ | <LLQ | <LLQ | <LLQ |
| <LLQ, less than the lower limit of quantification.  0 means the compound was not detected. | | | | | |

Table S3.3. Quantitative GCMS results for the Banagrass pyrolysis oils recovered at the second shortest residence time (BP-3) over four temperatures. Results are presented as wt% relative to feedstock (daf).

| **Target Compounds** | LLQ | Temperature / °C | | | |
| --- | --- | --- | --- | --- | --- |
|  |  | 400 | 450 | 500 | 600 |
|  | ug/mL | wt% | wt% | wt% | wt% |
| Cyclohexane | 5 | 0 | 0 | 0 | 0 |
| Furfural | 15 | <LLQ | <LLQ | <LLQ | <LLQ |
| 3-methyl-2-cyclopenten-1-one | 15 | <LLQ | <LLQ | <LLQ | 0 |
| Phenol | 5 | 0.16 | 0.08 | 0.16 | 0.17 |
| 2-methoxy-phenol | 10 | 0.17 | 0.20 | 0.17 | <LLQ |
| o-Cresol | 10 | <LLQ | <LLQ | <LLQ | <LLQ |
| p-Cresol | 10 | <LLQ | <LLQ | 0.15 | <LLQ |
| m-Cresol | 5 | 0.07 | 0.07 | 0.08 | 0.07 |
| Creosol | 5 | 0.19 | 0.19 | 0.08 | 0.14 |
| 2,4-Dimethyl-phenol | 5 | 0 | 0.16 | <LLQ | 0.14 |
| 4-ethyl-phenol, | 10 | 0.21 | 0.19 | 0.16 | 0.19 |
| 2,6-dimethoxy-phenol, | 5 | 0.23 | 0.23 | 0.19 | 0.20 |
| Indole | 5 | <LLQ | <LLQ | <LLQ | 0 |
| Isoeugenol | 10 | 0.39 | 0.39 | 0.26 | 0.38 |
| Benzene | 10 | 0 | 0 | <LLQ | <LLQ |
| Naphthalene | 10 | 0 | 0 | <LLQ | <LLQ |
| <LLQ, less than the lower limit of quantification.  0 means the compound was not detected. | | | | | |

Table S3.4. Quantitative GCMS results for the Banagrass pyrolysis oils recovered at the second shortest residence time (BP-4) over four temperatures. Results are presented as wt% relative to feedstock (daf).

| Target Compounds | LLQ | Temperature / °C | | | |
| --- | --- | --- | --- | --- | --- |
|  |  | 400 | 450 | 500 | 600 |
|  | ug/mL | wt% | wt% | wt% | wt% |
| Cyclohexane | 5 | 0 | 0 | 0 | 0 |
| Furfural | 15 | <LLQ | <LLQ | <LLQ | 0 |
| 3-methyl-2-cyclopenten-1-one | 15 | 0 | 0 | <LLQ | <LLQ |
| Phenol | 5 | 0.20 | 0.18 | 0.07 | <LLQ |
| 2-methoxy-phenol | 10 | <LLQ | <LLQ | <LLQ | <LLQ |
| o-Cresol | 10 | <LLQ | <LLQ | <LLQ | <LLQ |
| p-Cresol | 10 | <LLQ | <LLQ | <LLQ | <LLQ |
| m-Cresol | 5 | <LLQ | 0.08 | 0.08 | <LLQ |
| Creosol | 5 | <LLQ | <LLQ | <LLQ | 0 |
| 2,4-Dimethyl-phenol | 5 | 0.10 | <LLQ | <LLQ | <LLQ |
| 4-ethyl-phenol, | 10 | 0.15 | 0.19 | 0.21 | <LLQ |
| 2,6-dimethoxy-phenol, | 5 | <LLQ | 0.15 | <LLQ | <LLQ |
| Indole | 5 | <LLQ | <LLQ | <LLQ | <LLQ |
| Isoeugenol | 10 | <LLQ | <LLQ | <LLQ | <LLQ |
| Benzene | 10 | 0 | 0 | <LLQ | 0 |
| Naphthalene | 10 | <LLQ | <LLQ | <LLQ | <LLQ |
| <LLQ, less than the lower limit of quantification.  0 means the compound was not detected. | | | | | |

1. Steele, P.H., et al., *Method to upgrade bio-oils to fuel and bio-crude.*, 2013, Mississippi State University.
