# Supplementary material for: Fast Pyrolysis Behavior of Banagrass as a Function of Temperature and Volatiles Residence Time in a Fluidized Bed Reactor
Source: PLoS One. 2015 Aug 26;10(8):e0136511. doi: 10.1371/journal.pone.0136511 (PMC4550300; doi:10.1371/journal.pone.0136511)
Supplement: S4 File — (DOCX) [file pone.0136511.s004.docx]

**Supporting Information - Fast pyrolysis behavior of banagrass as a function of temperature and volatiles residence time in a fluidized bed reactor**

**S4 File. Temperature distributions across the bed and freeboard**

Table S4.1 to S4.4 present the temperature distributions across the bed (T1 and T2) and the freeboard (T3 to T5) when operating the pyrolysis reactor with different bed positions. The positions of the thermocouples are described in the experimental section of the manuscript. The temperatures displayed in Tables S4.1 to S4.4 are the values noted 5 minutes before feeding the fuel, the temperatures remained within +/- 10 °C of the stated values during the course of an experiment. A three zone furnace is used to heat the reactor with the temperature of each zone set to obtain a uniform as possible temperature across the bed and freeboard. When the reactor is set up for the shortest vapor residence time experiments (bed position BP-4) the bed is located between the top two zones of the furnace which makes it difficult to keep the freeboard at a similar temperature as the bed. To avoid having a dramatically lower temperature in the freeboard than in the bed it was necessary to work with a slightly higher bed temperature than when the bed was in lower positions.

Table S4.1. Temperature distribution across the bed and freeboard for the four different bed positions when operating at 400 °C.

| Thermocouple position | Bed position | | | |
| --- | --- | --- | --- | --- |
|  | BP-1 | BP-2 | BP-3 | BP-4 |
|  | °C | °C | °C | °C |
| T1 | 399 | 400 | 401 | 409 |
| T2 | 399 | 395 | 400 | 403 |
| T3 | 400 | 362 | 411 | 373 |
| T4 | 401 | 407 | 387 | n/a |
| T5 | 398 | 399 | 389 | 349 |
| n/a, not applicable - as when the bed is in its highest position (BP-4) thermocouple T4 is no longer in the freeboard of the reactor | | | | |

Table S4.2. Temperature distribution across the bed and freeboard for the four different bed positions when operating at 450 °C.

| Thermocouple position | Bed position | | | |
| --- | --- | --- | --- | --- |
|  | BP-1 | BP-2 | BP-3 | BP-4 |
|  | °C | °C | °C | °C |
| T1 | 453 | 456 | 459 | 466 |
| T2 | 452 | 444 | 453 | 458 |
| T3 | 451 | 414 | 460 | 418 |
| T4 | 451 | 467 | 433 | n/a |
| T5 | 452 | 449 | 434 | 394 |
| n/a, not applicable - as when the bed is in its highest position (BP-4) thermocouple T4 is no longer in the freeboard of the reactor | | | | |

Table S4.3. Temperature distribution across the bed and freeboard for the four different bed positions when operating at 500 °C.

| Thermocouple position | Bed position | | | |
| --- | --- | --- | --- | --- |
|  | BP-1 | BP-2 | BP-3 | BP-4 |
|  | °C | °C | °C | °C |
| T1 | 499 | 499 | 506 | 512 |
| T2 | 497 | 495 | 503 | 507 |
| T3 | 504 | 473 | 511 | 473 |
| T4 | 500 | 517 | 475 | n/a |
| T5 | 495 | 499 | 473 | 452 |
| n/a, not applicable - as when the bed is in its highest position (BP-4) thermocouple T4 is no longer in the freeboard of the reactor | | | | |

Table S4.4. Temperature distribution across the bed and freeboard for the four different bed positions when operating at 600 °C.

| Thermocouple position | Bed position | | | |
| --- | --- | --- | --- | --- |
|  | BP-1 | BP-2 | BP-3 | BP-4 |
|  | °C | °C | °C | °C |
| T1 | 600 | 613 | 610 | 620 |
| T2 | 599 | 601 | 605 | 608 |
| T3 | 604 | 579 | 613 | 568 |
| T4 | 601 | 612 | 562 | n/a |
| T5 | 599 | 598 | 561 | 538 |
| n/a, not applicable - as when the bed is in its highest position (BP-4) thermocouple T4 is no longer in the freeboard of the reactor | | | | |
