# Supplementary material for: Fast Pyrolysis Behavior of Banagrass as a Function of Temperature and Volatiles Residence Time in a Fluidized Bed Reactor
Source: PLoS One. 2015 Aug 26;10(8):e0136511. doi: 10.1371/journal.pone.0136511 (PMC4550300; doi:10.1371/journal.pone.0136511)
Supplement: S5 File — (DOCX) [file pone.0136511.s005.docx]

**Supporting Information - Fast pyrolysis behavior of banagrass as a function of temperature and volatiles residence time in a fluidized bed reactor**

**S5 File. Pyrolysis product yields (bio-oil, char and gas) - data tables**

Table S5.1. Oil, char and gas yields (wt% daf) from banagrass pyrolysis at 2nd longest

residence time (BP-2)

| Temperature | Bed Position | Dry Bio-Oil | Volatile Bio-Oil^#^ | Char_Org_* | ^CO CO_2_ CH_4_ H_2_ | Undetected** |
| --- | --- | --- | --- | --- | --- | --- |
| °C |  | wt% | wt% | wt% | wt% | wt% |
| 400 | BP-2 | 29.2 | <LLQ | 8.3 | 7.7 | 54.8 |
| 450 | BP-2 | 27.8 | <LLQ | 3.9 | 8.5 | 59.9 |
| 500 | BP-2 | 25.6 | <LLQ | 2.6 | 12.8 | 59.0 |
| 600 | BP-2 | 10.5 | <LLQ | 2.5 | 29.2 | 57.8 |
| <LLQ, less than the lower limit of quantification.  ^#^ Volatile bio-oil refers to the amount of bio-oil removed from the sample during rotary evaporation and is determined by analyzing the bio-oil solution by GCMS before drying and again after it is dried.  ^ Indicative values derived from on-line gas analysis.  * The bias in the char yield is estimated to be ≤±2 wt% (absolute), values are for the daf char  ** ^'^Undetected' is derived as: 100% - (dry bio-oil + volatile bio-oil + char + CO, CO_2_, CH_4_ and H_2_ yields). | | | | | | |

Table S5.2. Oil, char and gas yields (wt% daf) from banagrass pyrolysis at 2nd shortest

residence time (BP-3)

| Temperature | Bed Position | Dry Bio-Oil | Volatile Bio-Oil^#^ | Char_Org_* | ^CO CO_2_ CH_4_ H_2_ | Undetected** |
| --- | --- | --- | --- | --- | --- | --- |
| °C |  | wt% | wt% | wt% | wt% | wt% |
| 400 | BP-3 | 26.6 | 0.2 | 8.9 | 8.4 | 56.0 |
| 450 | BP-3 | 28.0 | 0.2 | 3.9 | 9.0 | 58.9 |
| 500 | BP-3 | 25.7 | 0.2 | 3.5 | 11.6 | 59.0 |
| 600 | BP-3 | 13.9 | 0.2 | 1.0 | 24.7 | 60.3 |
| <LLQ, less than the lower limit of quantification.  ^#^ Volatile bio-oil refers to the amount of bio-oil removed from the sample during rotary evaporation and is determined by analyzing the bio-oil solution by GCMS before drying and again after it is dried.  ^ Indicative values derived from on-line gas analysis.  * The bias in the char yield is estimated to be ≤±2 wt% (absolute), values are for the daf char  ** ^'^Undetected' is derived as: 100% - (dry bio-oil + volatile bio-oil + char + CO, CO_2_, CH_4_ and H_2_ yields). | | | | | | |

Table S5.3. Oil, char and gas yields (wt% daf) from banagrass pyrolysis at shortest residence time (BP-4)

| Temperature | Bed Position | Dry Bio-Oil | Volatile Bio-Oil^#^ | Char_Org_* | ^CO CO_2_ CH_4_ H_2_ | Undetected** |
| --- | --- | --- | --- | --- | --- | --- |
| °C |  | wt% | wt% | wt% | wt% | wt% |
| 400 | BP-4 | 28.5 | 0.2 | n/a | 7.9 | n/a |
| 450 | BP-4 | 36.7 | 0.2 | 3.0 | 7.8 | 52.5 |
| 500 | BP-4 | 30.2 | <LLQ | 3.0 | 9.2 | 57.6 |
| 600 | BP-4 | 20.9 | <LLQ | 2.1 | 14.7 | 62.3 |
| <LLQ, less than the lower limit of quantification.  ^#^ Volatile bio-oil refers to the amount of bio-oil removed from the sample during rotary evaporation and is determined by analyzing the bio-oil solution by GCMS before drying and again after it is dried.  ^ Indicative values derived from on-line gas analysis.  * The bias in the char yield is estimated to be ≤±2 wt% (absolute), values are for the daf char  ** ^'^Undetected' is derived as: 100% - (dry bio-oil + volatile bio-oil + char + CO, CO_2_, CH_4_ and H_2_ yields). | | | | | | |
