# Supplementary material for: Fast Pyrolysis Behavior of Banagrass as a Function of Temperature and Volatiles Residence Time in a Fluidized Bed Reactor
Source: PLoS One. 2015 Aug 26;10(8):e0136511. doi: 10.1371/journal.pone.0136511 (PMC4550300; doi:10.1371/journal.pone.0136511)
Supplement: S6 File — (DOCX) [file pone.0136511.s006.docx]

**Supporting Information - Fast pyrolysis behavior of banagrass as a function of temperature and volatiles residence time in a fluidized bed reactor**

**S6 File. Breakdown of the permanent gas yields**

A breakdown of the permanent gas data in terms of weight percent of CO, CO_2_, CH_4_ and H_2_ relative to the amount of feedstock (daf) as a function of temperature and vapor residence time is presented in Figure S1 and in Tables S6.1 to S6.4. The total amount of gas produced at each condition is reported in Supporting Information, Tables S6.1 to S6.4, as liters of gas per gram of feedstock (daf). The permanent gas data was derived from online gas analyzers which are not guaranteed to give quantitative results, as explained in the experimental section. The results therefore should only be considered as indicative. Nonetheless, the repeatability of the results was found to be good (see Figure S1). Note, it is assumed that all the oxygen in the gases is from the organic part of the sample.

**Figure S1. Permanent gas data (CO, CO_2_, CH_4_ and H_2_) from the pyrolysis of banagrass as a function of temperature and vapor residence time, presented as wt% of the daf feedstock (BP, bed position).** The standard deviation for the CO values is ≤1.0 wt% (absolute), for CO_2_ ≤0.5 wt%, for CH_4_ ≤0.2 wt% and for H_2_ ≤0.05 wt%.

The permanent gases produced from the pyrolysis of banagrass are in all cases dominated by CO, followed by CO_2_, then CH_4_ and H_2_. There is a clear increasing trend in the absolute amount of gas produced with increasing temperature. The low CO_2_ yield (~2-5 wt% relative to the daf feedstock) is promising in terms of energy recovery in a potentially integrated process.

At 400 °C, hydrogen accounts for less than 0.05 wt% of the daf feedstock across all the residence times examined. Differences start to emerge at 450 °C. At shorter residences times (BP-4 to BP-2) the amount of H_2_ remains <0.05 wt% with a significant increase to ~0.08 wt% H_2_ at the longest residence time (BP-1). At 500 °C the weight percentage of H_2_ steadily increases with residence time from ~0.03 wt% at the shortest residence time to ~0.2 wt% at the longest. At 600 °C the amount of hydrogen also increased with residence time with ~0.14 wt% at the shortest residence time incrementally increasing to ~0.6 wt% at the longest residence time.

The amount of CO is unaffected by residence time at 400 °C, accounting for ~5 wt% of the daf feedstock. At 450 °C there is ~5-6 wt% CO at shorter residence times (BP-4 to BP-2) and ~8 wt% at the longest RT (BP-1). At 500 °C the amount of CO incrementally increased from ~6.5 wt% at the shortest residence time to ~11 wt% at the longest. At 600 °C more CO is formed, with ~11 wt% at the shortest residence time increasing to ~27 wt% at the longest RT.

Methane shows trends similar to CO, although the absolute amounts are much lower. Over the temperature range of 400 to 500 °C and across all residence times the amount of CH_4_ was ≤1 wt%. At 600 °C the amount of CH4 increased from ~0.8 wt% at the shortest residence time to ~2 wt% at the longest.

For CO_2_, the amount is also fairly constant over the temperature range of 400 to 500 °C across all residence times, accounting for ~2-3 wt% of the daf feedstock. At 600 °C the amount of CO_2_ increases from 2.4 wt% at the shortest residence time to 5-6 wt% at longer residence times.

Table S6.1. Permanent gas data from the pyrolysis of banagrass at the longest residence time (BP-1), presented as wt% relative to the daf feedstock.

| Temp | CO | CO2 | CH4 | H2 | Total Producer Gas |
| --- | --- | --- | --- | --- | --- |
| °C | wt% | wt% | wt% | wt% | L/g daf |
| 400 | 5.3 | 2.6 | 0.2 | 0.02 | 0.07 |
| 450 | 8.4 | 2.9 | 0.6 | 0.08 | 0.11 |
| 500 | 11.3 | 2.8 | 0.7 | 0.20 | 0.15 |
| 600 | 27.4 | 4.8 | 2.1 | 0.60 | 0.38 |
| The relative standard deviation in the 'total producer gas' values is less than ± 4 %.  The standard deviation for the CO values is ≤±1.0 wt% (absolute), for CO_2_ ≤±0.5 wt%, for CH_4_ ≤±0.2 wt% and for H_2_ ≤±0.05 wt%. | | | | | |

Table S6.2. Permanent gas data from the pyrolysis of banagrass at the second longest residence time (BP-2), presented as wt% relative to the daf feedstock.

| Temp | CO | CO2 | CH4 | H2 | Total Producer Gas |
| --- | --- | --- | --- | --- | --- |
| °C | wt% | wt% | wt% | wt% | L/g daf |
| 400 | 5.1 | 2.4 | 0.2 | 0.01 | 0.06 |
| 450 | 6.0 | 2.1 | 0.3 | 0.01 | 0.07 |
| 500 | 9.2 | 3.0 | 0.6 | 0.09 | 0.12 |
| 600 | 21.4 | 5.6 | 1.8 | 0.42 | 0.30 |
| The relative standard deviation in the 'total producer gas' values is less than ± 4 %.  The standard deviation for the CO values is ≤±1.0 wt% (absolute), for CO_2_ ≤±0.5 wt%, for CH_4_ ≤±0.2 wt% and for H_2_ ≤±0.05 wt%. | | | | | |

Table S6.3. Permanent gas data from the pyrolysis of banagrass at the second shortest residence time (BP-3), presented as wt% relative to the daf feedstock.

| Temp | CO | CO2 | CH4 | H2 | Total Producer Gas |
| --- | --- | --- | --- | --- | --- |
| °C | wt% | wt% | wt% | wt% | L/g daf |
| 400 | 5.1 | 3.0 | 0.3 | 0.01 | 0.07 |
| 450 | 5.1 | 3.3 | 0.3 | 0.03 | 0.07 |
| 500 | 7.7 | 3.0 | 0.5 | 0.05 | 0.10 |
| 600 | 17.4 | 4.0 | 1.1 | 0.27 | 0.23 |
| The relative standard deviation in the 'total producer gas' values is less than ± 4 %.  The standard deviation for the CO values is ≤±1.0 wt% (absolute), for CO_2_ ≤±0.5 wt%, for CH_4_ ≤±0.2 wt% and for H_2_ ≤±0.05 wt%. | | | | | |

Table S6.4. Permanent gas data from the pyrolysis of banagrass at the shortest residence time (BP-4), presented as wt% relative to the daf feedstock.

| Temp | CO | CO2 | CH4 | H2 | Total Producer Gas |
| --- | --- | --- | --- | --- | --- |
| °C | wt% | wt% | wt% | wt% | L/g daf |
| 400 | 5.2 | 2.4 | 0.3 | 0.02 | 0.07 |
| 450 | 5.2 | 2.3 | 0.3 | 0.01 | 0.06 |
| 500 | 6.5 | 2.3 | 0.4 | 0.03 | 0.08 |
| 600 | 11.4 | 2.4 | 0.8 | 0.14 | 0.14 |
| The relative standard deviation in the 'total producer gas' values is less than ± 4 %.  The standard deviation for the CO values is ≤±1.0 wt% (absolute), for CO_2_ ≤±0.5 wt%, for CH_4_ ≤±0.2 wt% and for H_2_ ≤±0.05 wt%. | | | | | |
