# Supplementary figures and images for: Fast Pyrolysis Behavior of Banagrass as a Function of Temperature and Volatiles Residence Time in a Fluidized Bed Reactor
Source: PLoS One. 2015 Aug 26;10(8):e0136511. doi: 10.1371/journal.pone.0136511 (PMC4550300; doi:10.1371/journal.pone.0136511)

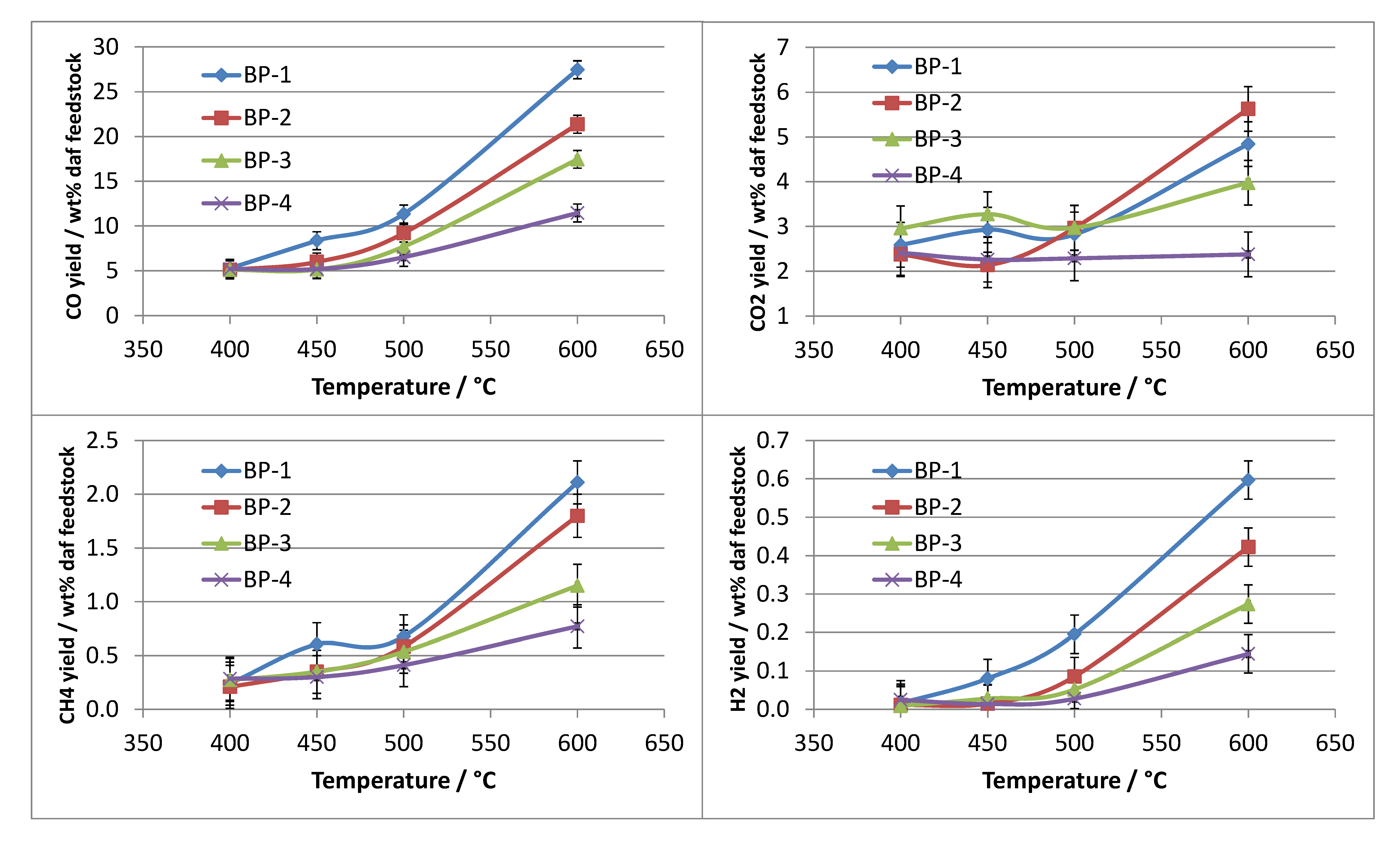

Supplement: S1 Fig — (TIF) [file pone.0136511.s007.tif]
